# *Phytochrome Interacting Factors* (*PIFs*) in *Solanum lycopersicum*: Diversity, Evolutionary History and Expression Profiling during Different Developmental Processes

**DOI:** 10.1371/journal.pone.0165929

**Published:** 2016-11-01

**Authors:** Daniele Rosado, Giovanna Gramegna, Aline Cruz, Bruno Silvestre Lira, Luciano Freschi, Nathalia de Setta, Magdalena Rossi

**Affiliations:** 1 Departamento de Botânica, Instituto de Biociências, Universidade de São Paulo, São Paulo, SP, Brazil; 2 Centro de Ciências Naturais e Humanas, Universidade Federal do ABC, Santo André, SP, Brazil; Instituto de Biologia Molecular y Celular de Plantas, SPAIN

## Abstract

Although the importance of light for tomato plant yield and edible fruit quality is well known, the PHYTOCHROME INTERACTING FACTORS (PIFs), main components of phytochrome-mediated light signal transduction, have been studied almost exclusively in *Arabidopsis thaliana*. Here, the diversity, evolution and expression profile of *PIF* gene subfamily in *Solanum lycopersicum* was characterized. Eight tomato *PIF loci* were identified, named *SlPIF1a*, *SlPIF1b*, *SlPIF3*, *SlPIF4*, *SlPIF7a*, *SlPIF7b*, *SlPIF8a* and *SlPIF8b*. The duplication of *SlPIF1*, *SlPIF7* and *SlPIF8* genes were dated and temporally coincided with the whole-genome triplication event that preceded tomato and potato divergence. Different patterns of mRNA accumulation in response to light treatments were observed during seedling deetiolation, dark-induced senescence, diel cycle and fruit ripening. *SlPIF4* showed similar expression profile as that reported for *A*. *thaliana* homologs, indicating an evolutionary conserved function of PIF4 clade. A comprehensive analysis of the evolutionary and transcriptional data allowed proposing that duplicated *SlPIFs* have undergone sub- and neofunctionalization at mRNA level, pinpointing the importance of transcriptional regulation for the maintenance of duplicated genes. Altogether, the results indicate that genome polyploidization and functional divergence have played a major role in diversification of the *Solanum PIF* gene subfamily.

## Introduction

Every aspect of plant physiology is influenced by light. Right after germination, etiolated growth (skotomorphogenesis) allows seedlings to seek for light at the soil surface and, upon light exposure, signal transduction initiates photomorphogenic development (deetiolation), characterized by chloroplast differentiation and initiation of photosynthetic activity. During autotrophic vegetative development, light provides the energy that fuels plant growth, designs architecture of mature plant and regulates flowering. Furthermore, light deprivation is an important senescence inducer in lower leaves shaded by upper leaves for nutrient remobilization. The capability to adjust to environmental light conditions is mediated by photoreceptors, which perceive and transduce light signals to the downstream transcriptional network that triggers adaptive responses [1].

*Solanum lycopersicum*, a fleshy fruit bearing species, is an excellent model for deciphering light signal transduction network. Firstly, because tomato plant yield and edible fruit quality are determined by plastid biogenesis and activity that, in turn, are highly dependent on light perception and transduction. *High pigment* tomato mutants, *hp1* and *hp2*, are deficient in the negative regulators of light signal transduction *DAMAGE DNA BINDING PROTEIN 1* (*DDB1*) and *DE-ETIOLATED* (*DET1*), respectively. The fruits of these plants show increased levels of chlorophyll and higher levels of the nutraceutical carotenoids, flavonoids and tocopherols in immature and mature stages, respectively [2,3]. Light-grown seedlings of tomato transgenic lines silenced for *ELONGATED HYPOCOTYL 5* (*HY5*), a positive regulator of light signaling involved in plastid biogenesis, displayed etiolated phenotype and adult plants showed over 30% reduction in leaf and immature fruit chlorophyll accumulation. Moreover, total carotenoid levels in ripe fruits of HY5-deficient plants were significantly decreased compared to wild type controls [4]. Secondly, *Solanum* lineage have been affected by two whole-genome triplications; the first occurred before the divergence between *Arabidopsis* and *Solanum* more than 120 MYA, while the second preceded the divergence between tomato and potato estimated at 71 (± 19.4) MYA [5]. Polyploidization events provide the basis for the evolution of novel functions and, in particular, the expansion of genes encoding transcription factors correlates with the evolutionary gain of morphological complexity [6]. In this sense, it has been proposed that these genome triplications contributed with fruit-specific functions in tomato, such as the ripening master transcription factor *RIPENING INHIBITOR* (*RIN*) and phytochrome (PHYs) photoreceptors that influence fruit quality [5].

PHYs are major photoreceptors that perceive red (R)/far-red (FR)-light. Five PHYs *loci* have been identified in tomato genome designated *PHYA*, *PHYB1*, *PHYB2*, *PHYE* and *PHYF* in accordance to the *A*. *thaliana PHYA* to *PHYE* homologs [7]. The role of the tomato *PHYs* in vegetative development has been explored by the characterization of mutants [8] and overexpressing [9] plants for *PHYA*, *PHYB1* and *PHYB2*. Increasing *PHYA* and *PHYB1* expression rendered mild effects on anthocyanin levels and on seedling and adult plant development. On the contrary, transgenic plants with high levels of *PHYB2* showed an acute inhibition of elongation, enhancement of anthocyanin accumulation, and strong amplification of the red light high irradiance response [9]. By using single, double or triple mutants (*phyA*, *phyB1*, *phyB2*, *phyB1B2*, *phyAB1* and *phyAB1B2*), a recent report evaluated the participation of different phytochrome species in the regulation of fruit development and ripening. The results showed that the impairment in distinct *PHYs* differentially influences the time intervals among fruit developmental stages as well as the carotenoid content [10].

PHYs exist in two different forms, the R-absorbing Pr form and the FR-absorbing Pfr form. R triggers activation of PHYs by converting the Pr form to the Pfr form, whereas FR inactivates Pfr converting it back to the Pr form. Active PHYs Pfr form is translocated to the cell nucleus where it physically interacts with the PHYTOCHROME-INTERACTING FACTORS (PIFs). PIFs are basic helix–loop–helix (bHLH) transcription factors that play a key role in PHY-mediated light signal transduction being part of the regulatory network of a wide range of developmental processes, from seed germination towards senescence. However, with few exceptions [11–14], *PIFs* have been only studied in *A*. *thaliana*. PIF proteins have an Active Phytochrome B-binding (APB) and a DNA-binding bHLH domain. The canonical PIFs, *i*.*e*. PIF1, PIF3, PIF4, PIF5 and PIF7, physically interact with PHYB; while PIF1 and PIF3 also interact with PHYA through an Active Phytochrome A-binding (APA) domain. Pfr-PIF interaction triggers phosphorylation and subsequent proteasomal degradation of PIFs, which leads to physiological responses. A notable exception to this dynamic behavior is PIF7, which despite interacting with PHYB shows no detectable light-induced degradation [1]. Several target genes for *A*. *thaliana* PIF proteins have been identified. PIF3 mediates the initial phases of seedling light-induced chloroplast development during deetiolation through the regulation of nuclear genes involved in photosynthesis and chloroplast biogenesis [15]. ChIP–PCR experiment confirmed that PIF4 binds to the E-box motifs of the promoters of both chloroplast activity maintainer genes *GOLDEN 2-LIKE 1* (*GLK1*) and *GLK2*, repressing their expression [16]. Additionally, PIF4 and PIF5 act as transcriptional activators of the master senescence transcription factor *ORESARA 1* (*ORE1*) and chlorophyll degrading enzyme encoding genes, such as *STAY GREEN 1* (*SGR1*) and *NON-YELLOW COLORING 1* (*NYC1*), during dark-induced senescence by direct interaction with the G-box motifs on the corresponding promoter regions [16–18]. Finally, PIF1 has been shown to directly bind the G-box motif of the promoter of the chlorophyll and carotenoid biosynthetic genes *PROTOCHLOROPHYLLIDE OXIDOREDUCTASE* and *PHYTOENE SYNTHASE* (*PSY*), inducing and inhibiting their transcription, respectively [19,20]. Only one tomato *PIF* gene has been characterized so far, *PIF1a*, and, in agreement with its *Arabidopsis* ortholog showed to modulate carotenoid biosynthesis during fruit ripening. During green stages of fruit development, as a consequence of self-shading, Chl reduces R/FR ratio stabilizing PIF1a, which, in turn, represses the expression of the fruit-specific *PSY1*. After the onset of ripening, degreening allows the activation of Pfr and the consequent PIF1a degradation releases *PSY1* transcription, enhancing carotenogenesis [12, 21].

Considering the importance of light perception and signaling for plant development and fruit quality and, the poorly available knowledge about *PIF* genes in tomato; here we performed a comprehensive characterization of this gene subfamily in *S*. *lycopersicum*. By surveying the tomato genome, we identified eight *PIF* homolog sequences. The phylogenetic, divergence time estimation and selective pressure evaluation analyses allowed us to reconstruct the evolutionary history of *PIF* genes in *S*. *lycopersicum* and closely related Solanaceae species, the wild tomato *S*. *pennellii* and *S*. *tuberosum*. We further explored the transcriptional profile in four different developmental contexts, deetiolation, dark-induced senescence, daily cycle and fruit ripening, and identified expression patterns that suggest functional specificity. The data were discussed in the context of tomato genome evolution.

## Results

### Phylogenetic and Evolutionary Analysis of PIF *loci*

**Fig 1:**
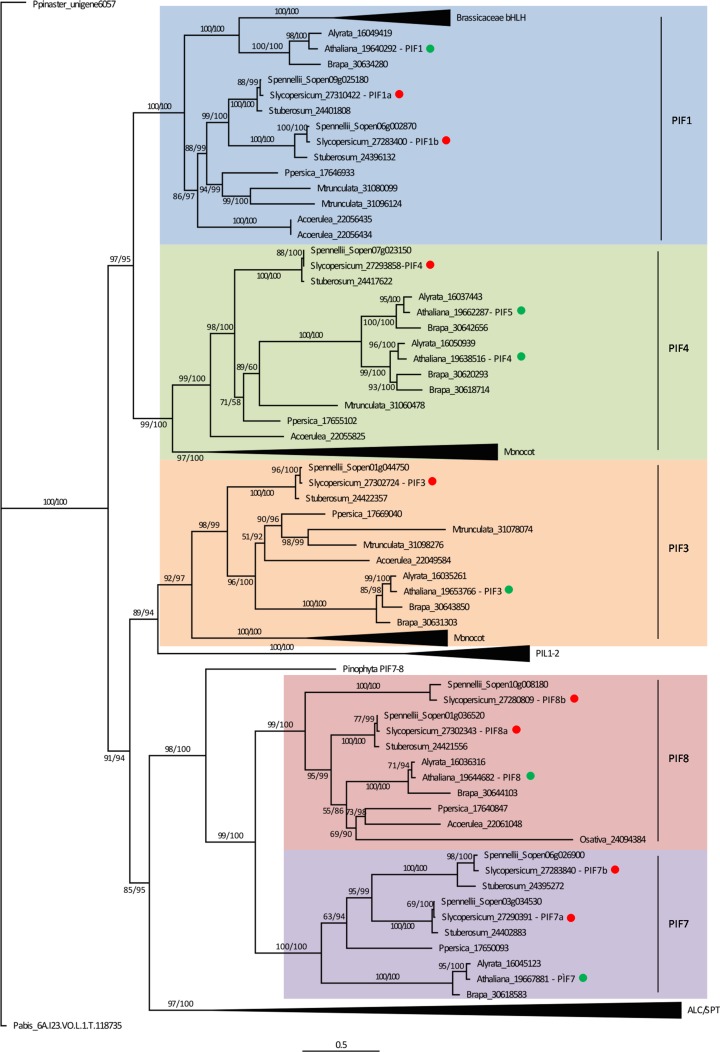
Phylogenetic reconstruction of PIF protein family. Phylogenetic analysis of PIF protein subfamily in Viridiplantae performed with 112 sequences from 13 species. Accession numbers of all sequences are detailed in S1 Table. Compacted clades encompassing more than one sequence are indicated by black triangles. *Arabidopsis thaliana* and *Solanum lycopersicum* sequences are indicated with green and red circles, respectively. PIF clades are highlighted with colored squares. Numbers at nodes represent bootstrap/approximate likelihood-ratio test (aLRT) values.

By performing a BLAST search against fully sequenced genome databases using *A*. *thaliana* canonical PIF sequences as queries, 119 sequences from 16 species were retrieved including sequences of the bHLH superfamily that do not belong to the PIF subfamily [1] (see Material and Methods, S1 Table). In agreement with previous report, no PIF homologs were found in chlorophytes [22]. In the basal land plants *Marchantia polymorpha* (liverworth), *Physcomitrella patens* (moss) and *Selaginella moellendorffii* (lycophyte), one, four and three *PIF* homologs were identified, respectively. Spermatophyte species harbor several gene copies that, based on the phylogenetic reconstruction, are mainly divided in two super clades named according to the corresponding *A*. *thaliana* homolog representative. The first contains PIF1 and PIF4 sequences and, the second encompasses PIF3, PIF3-like 1 and 2 (PIL1/2) [23], PIF8, PIF7, *ALCATRAZ* (*ALC*) and *SPATULA* (*SPT*) [24] sequences. In the second clade, PIF3 and PIL1/2, PIF7 and PIF8 and, ALC and SPT clustered together, respectively (Fig 1, S1 Fig, S1 Text).

Whereas *Arabidopsis* has six PIF encoding genes, henceforth named *AtPIFs*, eight *loci* were identified in *S*. *lycopersicum* genome, corresponding to the following accessions in Sol Genomics Network database [25]: *SlPIF1a*: Solyc09g063010, *SlPIF1b*: Solyc06g008030, *SlPIF3*: Solyc01g102300, *SlPIF4*: Solyc07g043580, *SlPIF7a*: Solyc03g115540, *SlPIF7b*: Solyc06g069600, *SlPIF8a*: Solyc01g090790, *SlPIF8b*: Solyc10g018510 (Fig 1). Aminoacid pairwise sequence alignments indicated that *Arabidopsis* and tomato homologs share 27–51% identity (S2 Table). Despite this low identity score, the APB-binding and bHLH domains were found in all tomato protein sequences, reinforcing their identity as PIF proteins. However, it is worth mentioning that tomato SlPIF1b, SlPIF4 and SlPIF8b display an amino acid substitution in the APB-binding domain that alters the conserved Q residue to G, E and E, respectively [26]. On the contrary, APA-binding domain was exclusively identified in *SlPIF1s* and *SlPIF3* (S2 Fig). Interestingly, the tree topology clearly showed that *Arabidopsis AtPIF4* and *AtPIF5* genes were originated by a Brassicaceae exclusive duplication, explaining the existence of a single gene in tomato genome within the clade PIF4. No differences in gene copy number were observed between *S*. *lycopersicum* and the most distantly related species within Lycopersicon section (*i*.*e*. tomatoes), *S*. *pennellii*. For PIF1, PIF7 and PIF8 clades, the analyzed tomato species harbor two gene copies, while for PIF3 and PIF4 a single copy was identified. *S*. *tuberosum* has a similar *PIF* gene copy number, excepting for a single *PIF8 locus* (Fig 1).

**Fig 2:**
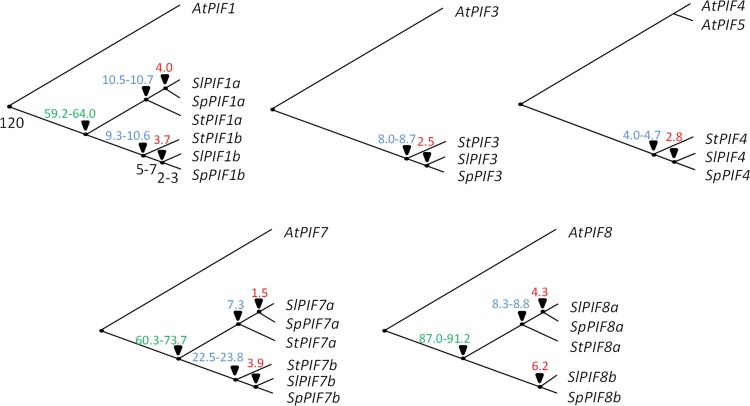
Divergence time estimations for *PIF* genes. The divergence times between the duplicated *PIF* genes in Solanaceae are shown in green. The divergence times between tomatoes (*S*. *lycopersicum* and *S*. *pennellii*) and *S*. *tuberosum* and, *S*. *lycopersicum* and *S*. *pennellii* homologs are indicated in blue and red, respectively. Species divergence times are shown in black (*Arabidopsis thaliana*-Solanaceae [34], *Solanum tuberosum*- *Solanum lycopersicum* [30], *S*. *pennelli*-*S*. *lycopersicum* [27]). Values are expressed in million years ago.

To gain insight on the evolutionary history of *PIF* gene family, we estimated the divergence time of *PIFs* using molecular clock [27]. The duplication of *PIF1*, *PIF7* and *PIF8* was estimated in a range of time from 59.2 to 91.2 MYA (millions of years ago). As expected [28,29], our data indicated that tomato and potato *PIF* genes diverged around the species splitting event (Fig 2) estimated about 5.1 to 7.3 MYA [30]; excepting *PIF7b*, for which an estimate of 22.5–23.8 MYA was retrieved. Similarly, the divergence of *S*. *lycopersicum* and *S*. *pennellii PIF* genes dates close to the estimated age of the most recent common ancestors within the species, 2.2–3.1 MYA [27], with the exception of *PIF8b*, for which a value of 6.2 MYA was obtained. The high divergence times observed for *PIF7b* and *PIF8b* are consequence of high synonymous substitution values (*dS*). Aiming to test whether the high *dS* values were consequence of positive selection or neutral evolution, we evaluated the selective constraints under which *PIF* gene are evolving (Table 1). Indeed, *PIF7b* showed signatures of positive selection, particularly in threonine 343 and serine 369 (BEB test *P*>95%). The rest of the *PIF* clades showed to be evolving under purifying selection. Unfortunately, we were unable to perform the test for *PIF8b* because it is absent in *S*. *tuberosum*.

**Table 1:** Evolutionary analysis of Solanaceae *PIF* genes.

		*PIF1a*	*PIF1b*	*PIF3*	*PIF4*	*PIF7a*	*PIF7b*	*PIF8a*
**Number of sequences**		3	3	3	3	3	3	3
**Number of codons**		552	499	702	495	435	397	461
**Mean dN**^**a**^ **± SD**		0.02±0.01	0.05±0.03	0.02±0.01	0.01±0.01	0.01±0.01	0.06±0.04	0.02±0.01
**Mean dS**^**b**^ **± SD**		0.08±0.04	0.08±0.04	0.07±0.03	0.04±0.01	0.05±0.03	0.16±0.11	0.07±0.02
**Mean Nc**^**c**^ **± SD**		55.03±0.80	54.38±0.45	50.21±0.73	51.41±0.31	53.08±0.53	54.03±0.52	59.20±0.42
**M0**^**d**^	**ω0**^**g**^	0.29	0.73	0.45	0.28	0.36	0.42	0.26
	**p0**^**h**^	1.00	1.00	1.00	1.00	1.00	1.00	1.00
	**lnl**^**i**^	-2752.11	-2668.30	-3471.89	-2250.83	-2047.17	-2327.67	-2241.83
**M1a**^**e**^	**ω0**	0.29	0.00	0.00	0.00	0.36	0.00	0.19
	**p0**	1.00	0.36	0.57	0.73	1.00	0.58	0.91
	**ω1**^**j**^	1.00	1.00	1.00	1.00	1.00	1.00	1.00
	**p1**^**k**^	0.00	0.64	0.43	0.27	0.00	0.42	0.09
	**lnl**	-2752.11	-2666.07	-3469.56	-2249.99	-2047.17	-2318.59	-2241.72
**M2a**^**f**^	**ω0**	0.29	0.00	0.00	0.00	0.36	0.14	0.19
	**p0**	1.00	0.59	0.69	0.79	1.00	0.69	0.91
	**ω1**	1.00	1.00	1.00	1.00	1.00	1.00	1.00
	**p1**	0.00	0.00	0.00	0.17	0.00	0.29	0.06
	**ω2**^**l**^	1.00	1.94	1.51	2.62	1.00	19.48	1.00
	**p2**^**m**^	0.00	0.41	0.31	0.05	0.00	0.02	0.03
	**lnl**	-2752.11	-2664.59	-3469.21	-2249.89	-2047.17	-2313.73	-2241.72
**2Δl (M1a-M0)**^**n**^		0	4.44	4.66	1.7	0	18.15 **	0.24
**2Δl (M2a-M1a)**^**o**^		0	2.98	0.7	0.19	0	9.72*	0

**a** dN **a** dN: non-synonymous distances and the corresponding standard deviation (SD)

**b** dS: synonymous distances and the corresponding standard deviation (SD)

**c** Nc: effective number of codons and the corresponding standard deviation (SD)

**d** M0: the null hypothesis, one-ratio model

**e** M1a: nearly neutral model

**f** M2a: positive selection model

**g** ω0: ω estimates for the codons under purifying selection

**h** p0: estimated proportion of codons under purifying selection

**i** lnl: log likelihood of model

**j** ω1: ω estimates for the codons under neutral evolution

**k** p1: estimated proportion of codons under neutral evolution

**l** ω2: ω estimates for codons under positive selection

**m** p2: estimated proportion of codons under positive selection

**n** 2Δl (M1a–M0): the likelihood ratio statistics (2Δl) is approximated by the χ2 distribution (degree of freedom = 1), null hypothesis (M0) rejected is highlighted in bold

**o** 2Δl (M2a-M1a): the likelihood ratio statistics (2Δl) is approximated by the χ2 distribution (degree of freedom = 2), M1a rejected is highlighted in bold. Single and double asterisk indicate *P*<0.01 and *P*<0.001, respectively.

### *PIF* Transcript Profile along Seedling Deetiolation, Daily Cycle, Dark-Induced Senescence and Fruit Ripening

*A*. *thaliana* PIF proteins are known regulators of seedling deetiolation and dark-induced senescence, and are modulated by the circadian clock [1,17]. Particularly in *S*. *lycopersicum*, recently, the role of *SlPIF1a* in ripening-associated carotenogenesis was also been demonstrated [12]. To evaluate the functional diversity of tomato *PIF* genes, a comprehensive mRNA accumulation profiling was performed during seedling deetiolation, dark-induced senescence, diel cycle and fruit ripening. *SlPIF8a* and *SlPIF8b* were not considered for functional analyses because there are no publications demonstrating *A*. *thaliana* PIF8 and PHYs interaction, therefore, this clade was not considered a canonical PIF.

**Fig 3:**
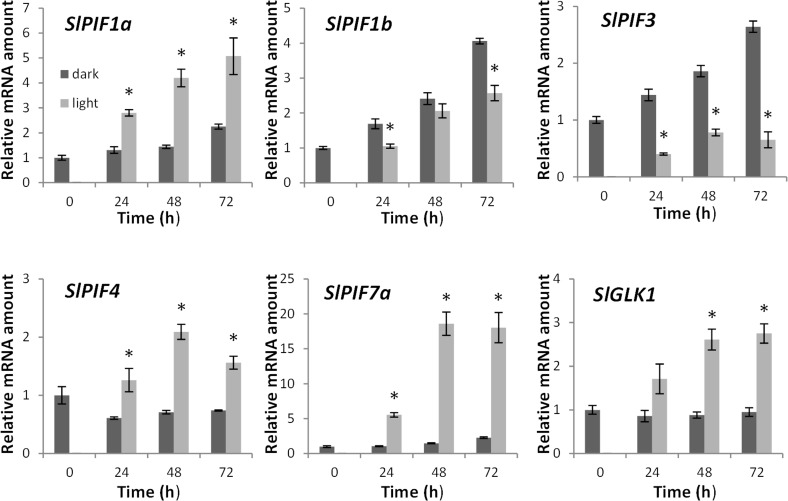
Expression profile of *PIF* genes in cotyledons under contrasting light conditions. Seedlings were grown in dark for 4 days and were either kept in darkness or transferred to continuous white light treatment. Significant differences (P<0,05) among treatments are indicated by asterisks. Values shown are means ± SE of at least three biological replicates.

The expression profile of *SlPIF* genes during deetiolation was analyzed in 4 day-old dark-grown seedlings exposed to 24, 48 and 72 h of constant light or dark conditions. The dark-treated seedlings exhibited typical skotomorphogenic phenotype presenting long hypocotyls as well as closed, small and chlorotic cotyledons. Seedlings exposed to constant light underwent photomorphogenesis and showed shorter hypocotyls, opened apical hooks, expanded and green cotyledons and anthocyanin accumulation (S3A Fig). Cotyledon chlorophyll accumulation (S3B Fig) and mRNA levels of the chloroplast activity maintainer gene *SlGLK1* [31] (Fig 3) confirmed the skotomorphogenetic and the photomorphogenetic growth of the dark and light-treated seedlings, respectively. Light induced the expression of *SlPIF1a*, *SlPIF4* and *SlPIF7a*, whereas *SlPIF1b* and *SlPIF3* mRNA levels were significantly reduced upon light exposure. Interestingly, *SlPIF1* paralogs showed contrasting patterns of light regulation displaying an increase up to 5-fold for *SlPIF1a* and 4-fold for *SlPIF1b* after 72 h of light and dark treatment, respectively. No significant transcript levels of *SlPIF7b* were detected in either treatment. A similar expression pattern of *SlPIF* genes was observed in the hypocotyls of light- and dark-treated seedlings (S3C Fig). It is worth noting that in terms of relative expression, *SlPIF1b* was the most abundantly *PIF* gene expressed in seedlings, both in cotyledons and hypocotyls. In cotyledons, *SlPIF4* showed intermediate mRNA levels followed by *SlPIF1a*, *SlPIF3* and *SlPIF7a*. In hypocotyls, *SlPIF4*, *SlPIF1a* and *SlPIF3* displayed similar intermediate amounts of transcript, while *SlPIF7a* was the least abundantly expressed (S3 Table).

**Fig 4:**
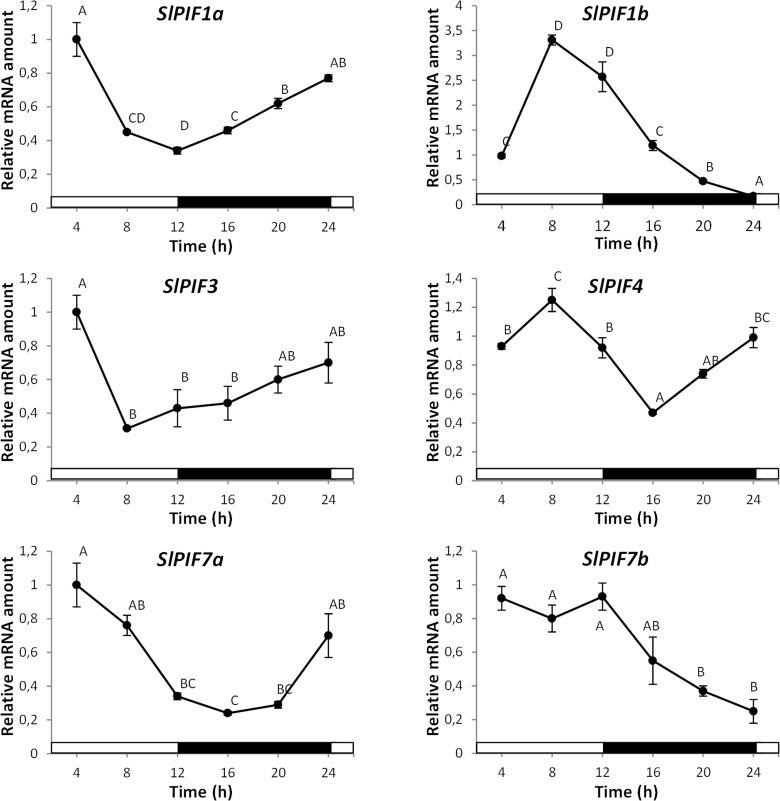
Expression profile of *PIF* genes during daily cycle. 3-week-old plants were grown under 12 h/12h light/dark photoperiod. The second fully expanded leaves were harvested every 4 h. White and black bars represent light and dark periods, respectively. Different letters indicate statistical differences (P<0.05). Values shown are means ± SE of at least three biological replicates.

The expression pattern of tomato *PIFs* during 24 h under 12/12 light/dark photoperiod was analyzed in 3-week-old plants (Fig 4). *SlPIF1a*, *SlPIF3* and *SlPIF7a* showed similar oscillation patterns, characterized by lowest transcript abundance at the end of the light period followed by a progressive increase during the dark period and maximum levels 4 h after dawn. *SlPIF4* mRNA abundance was significantly reduced during the afternoon achieving the lowest level at dusk and progressively increasing over the night to reach the maximum level 8 h after dawn. *SlPIF7b* mRNA levels were high and constant during the light period, progressively decreasing during the night. *SlPIF1a* and its paralog *SlPIF1b* exhibited distinct diel expression patterns since during the night period *SlPIF1a* and *SlPIF1b* mRNA levels progressively increased and decreased, respectively. Interestingly, *SlPIF1* genes were the most copiously expressed *PIF*s at beginning of the light period displaying over 2-fold more transcripts in leaves than the other *PIF* genes (S3 Table).

**Fig 5:**
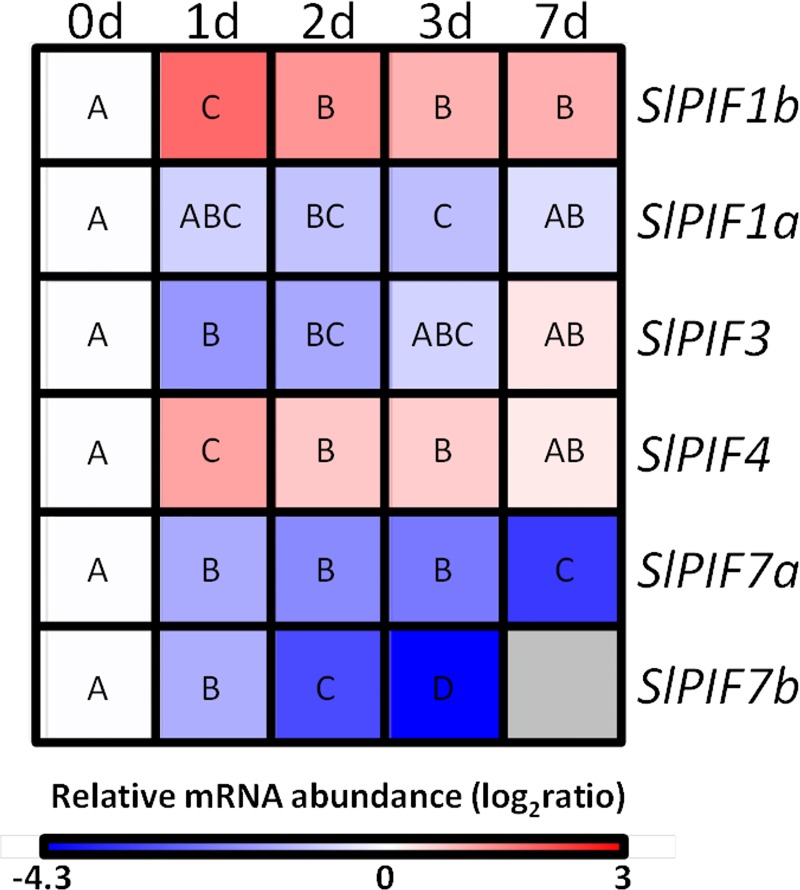
Expression profile of *PIF* genes during dark-induced senescence. 3-week-old plants were grown under 12 h/12 h light/dark photoperiod and transferred to constant darkness during 7 d and the second fully expanded leaves was sampled every day 4 h after the beginning of the light period. Heatmap representation of the relative mRNA abundance compared to day 0. Different letters indicate statistical differences (P<0.05). Values shown are means ± SE of at least three biological replicates.

Furthermore, we explored the transcriptional profile of *S*. *lycopersicum PIF* genes in leaves sampled from 3-week-old-plants maintained in darkness for 0, 1, 2, 3 and 7 days. The leaves showed clear signs of senescence as evidenced by the reduction in chlorophyll content at the seventh day (S4A Fig). The degreening was explained by the increment in *PHEOPHYTINASE* expression, the enzyme responsible for chlorophyll dephytylation in tomato leaves [32] and accompanied by a reduction in *SlGLK1* transcripts. Additionally, the induction of senescence was confirmed by the mRNA accumulation of the senescence marker *SENESCENCE-ASSOCIATED GENE 12* (*SlSAG12*, [33]) and *A*. *thaliana ORE1* homologs *SlORE1S02*, *SlORE1S03* and *SlORE1S06* (S4B Fig). These data allowed us to conclude that after 7 days of dark treatment, the plants underwent dark-induced senescence. Transcriptional profiling revealed that *SlPIF1a*, *SlPIF3*, *SlPIF7a* and *SlPIF7b* were downregulated whereas *SlPIF1b* and *SlPIF4* were upregulated upon darkness exposure, suggesting that probably the formers are involved in dark-induced senescence signaling (Fig 5).

**Fig 6:**
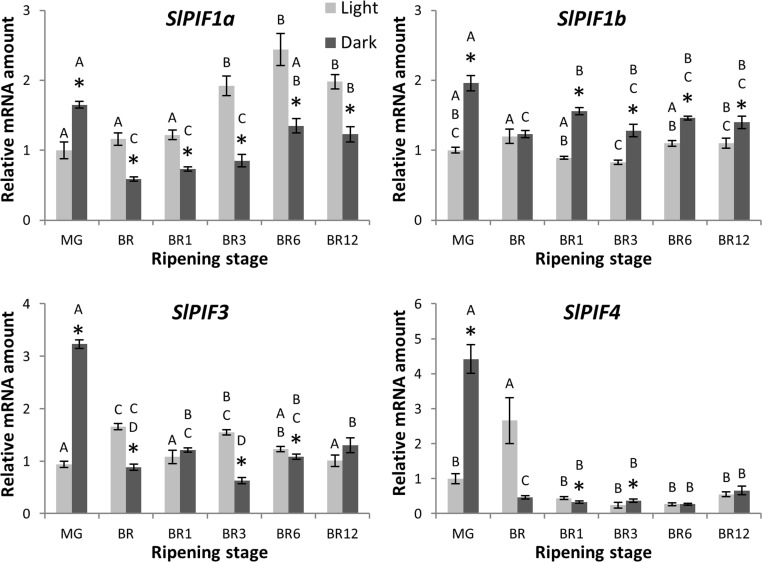
Expression profile of PIF genes during ripening under contrasting light conditions. Fruits were harvested at MG (mature-green) stage and left to ripen under constant light or dark conditions. Pericarp samples were harvested at MG (two days after the beginning of treatment), BR (breaker), BR1 (1 day after BR), BR3, BR6 and BR12 stages. Asterisks and letters represent significant (P<0.05) differences between treatments and stages, respectively. Values shown are means ± SE of at least three biological replicates.

To address the transcriptional behavior of all six *SlPIF* genes during tomato fruit ripening and evaluate their possible involvement in the light-dependent regulation of this developmental process, fruits at mature-green (MG) stage were harvested and left ripen under constant light or dark conditions. Total chlorophyll and carotenoids levels were measured and, as expected a concomitant reduction in total chlorophylls temporally coincided with the accumulation of the main carotenoids typically found in tomato fruits, thereby demonstrating that the detached fruits were undergoing normal ripening (S5 Fig). The levels of transcripts for both *SlPIF7* paralogs were undetectable in all fruit stages analyzed. Under dark conditions, *SlPIF1a*, *SlPIF1b*, *SlPIF3* and *SlPIF4* mRNA levels peaked 2 days after the start of the treatment, when the fruits were still in MG stage, followed by a reduction at the breaker (BR) stage (Fig 6). During the progression of ripening, *SlPIF1a* showed to be transcriptionally induced and significantly higher in the presence of light, whereas the mRNA levels of its paralog, *SlPIF1b*, were clearly lower in light- than in dark-treated fruits and did not respond to ripening. Finally, *SlPIF3* and *SlPIF4* mRNA levels were relatively constantly low along ripening and did not show clear patterns of regulation by light and dark treatments. In terms of relative expression, the most abundantly expressed *PIF* gene in fruits was *SlPIF3*, with 17-, 6- and 1.7-fold more mRNA amount than *SlPIF4*, *SlPIF1a* and *SlPIF1b*, respectively (S3 Table).

## Discussion

The key role played by light signaling on tomato plant development and fruit nutritional value has been widely studied by the use of mutants and transgenic approaches [3,4]. However, *PIF* genes have been lagged behind and almost exclusively studied in *A*. *thaliana* [11–14]. Besides the well described PHY-mediated proteasomal degradation mentioned above, *PIF* genes are under tight transcriptional regulation as indicated by database and genome-wide binding site analyses for several *A*. *thaliana* transcription factors. Moreover, it has been suggested that AtPIFs regulate their own expression by a complex autoregulatory mechanism [34], pinpointing the importance of studies approaching the expression regulation at transcriptional level. Aiming to gain insights on the function of these genes in tomato, here we explored their genetic diversity and expression profiling along different physiological contexts.

*PIF* is one of the 26 gene subfamilies of the monophyletic bHLH superfamily of plant transcription factors [22]. By using the five well described PIF proteins sequences from *A*. *thaliana* as baits (*i*.*e*. AtPIF1/3/4/5/7), we retrieved 119 homologs from 16 species and performed a phylogenetic analysis that allowed the identification of eight clades in Spermatophyte species (Fig 1). A comparative study between tomato and grape genomes proposed that a whole-genome triplication affecting Rosids, which includes *Arabidopsis*, and Euasterids, which includes *Solanum*, occurred in a common eudicot ancestor more than 120 MYA [5,35]. Interestingly, the monocot representatives of our phylogenetic reconstruction, *Oryza sativa* and *Sorghum bicolor*, did not show *PIF* genes in all the eight identified clades (Fig 1, S1 Table). Further, another triplication estimated at 71 (± 19.4) MYA occurred in the *Solanum* lineage followed by widespread gene loss that predates the 7.3 MYA tomato–potato divergence [5,30]. This second event was likely the origin of the duplications within PIF1, PIF7 and PIF8 clades since the estimated divergence time between the duplicated genes coincided with the date of the whole-genome triplication (Fig 2). To confirm this hypothesis, the gene collinearity was analyzed along the flanking genomic regions of the duplicated genes. As demonstrated for Solanaceae *PSY* genes [5], the *SlPIF1*, *SlPIF7* and *SlPIF8* paralog regions showed recognizable small scale synteny (S6 Fig). Thus, these polyploidization events may represent the foundation of the PIF subfamily diversification.

The evolutionary history of a genome is the result of the interdependent diversification of different genetic features like regulatory sequences, mobile elements and coding regions. Although, it is expectable that the gene divergence time approximately coincides with the corresponding species splitting date, heterogeneity in the nucleotide substitution rates among genetic features within the genome can defy the molecular clock approach [36]. In this sense, the estimated divergence time for tomato and potato *PIF7b* genes significantly predated the splitting date between species; while a similar situation was observed for *S*. *lycopersicum* and *S*. *pennellii PIF8b*. Interestingly, the evolutionary analysis for *PIF7b* demonstrated signatures of positive selection, which can be associated to functional divergence. The absence of *PIF8b* in *S*. *tuberosum* might be attributed to stochastic gene loss; consequently, no evolutionary analysis was performed.

To explore the functional diversification of tomato *PIF* genes, a comprehensive expression profile was carried out under various physiological processes induced or regulated by light, such as deetiolation, daily cycle, senescence and fruit ripening. Interestingly, *SlPIF* genes displayed differential mRNA accumulation pattern at least along one of the analyzed contexts, suggesting that these genes have undergone functional specification. Little and fragmented information is currently available about the transcriptional regulation of *PIF* genes and only punctual similarities with our experimental conditions were found in literature. The transcription of *AtPIF4* and *AtPIF5* has shown to be upregulated in *Arabidopsis* seedlings upon white light exposure [37]. This result is consistent with our observation that tomato *SlPIF4* mRNA levels increase during deetiolation (Fig 3). Tomato *SlPIF4* and *SlPIF7a* transcript accumulation patterns during diel cycle resemble those observed for *Arabidopsis AtPIF4*, *AtPIF5 and AtPIF7*, whose mRNA levels are regulated by the circadian clock [38,39]. However, while *AtPIF1* and *AtPIF3* mRNA levels in *Arabidopsis* remained relatively constant along the diel cycle [37], the tomato *SlPIF1a*, *SlPIF1b* and *SlPIF3* oscillated under 12 h/12 h light/dark photoperiod. *SlPIF7b* transcript levels also fluctuated during the diel cycle, suggesting that all tomato *PIFs* are transcriptionally regulated by the circadian clock (Fig 4). Besides the diel cycling, *SlPIF7s* have shown to be exclusively expressed in true leaves (S3 Table), reinforcing their role in circadian response regulation as demonstrated for *AtPIF7* ortholog [40]. Similarities were also found with *Arabidopsis* during dark-induced senescence. In *Arabidopsis*, *AtPIF4* and *AtPIF5* exhibited a peak of transcript accumulation in leaves one day after dark treatment [16] triggering senescence through the activation of the master transcription factor *AtORE1* [17]. Accordingly, tomato *SlPIF4* mRNA reached the highest levels one day after the start of the constant dark treatment (Fig 4). Interestingly, tomato *ORE1* homologs, *SlORE1S02*, *SlORE1S03* and *SlORE1S06* were also induced by the dark treatment, suggesting that a similar functional link between *PIF4* and *ORE* genes regulates dark-induced senescence in both *Arabidopsis* and tomato (S4B Fig). A recent publication functionally characterized tomato *SlPIF1a* demonstrating its involvement as a negative regulator of fruit carotenogenesis [12]. Llorente et al. (2016) reported that *SlPIF1a* expression is induced along ripening and *SlPIF1b* is not expressed in fruits. Our data also showed that *SlPIF1a* transcripts do accumulate during ripening, however, the amount of *SlPIF1b* mRNA at MG stage was 3-fold higher than *SlPIF1a* (S3 Table, Fig 6). These apparent contrasting data might be the results of differences in experimental design, since the transcriptional profile showed here was performed from fruits ripened off vine and under constant light/dark treatments. Moreover, *SlPIF1a* transcript accumulated at higher levels in fruits ripened under light, while *SlPIF1b* transcription was repressed by this treatment. This opposite pattern of light response between *SlPIF1* duplicated genes was also observed in the other physiological contexts analyzed in this work and might be the result from differences in transcriptional promoter activities. Therefore, we surveyed a fragment of 2 kb upstream the translation initiation site of these genes by a *de novo* search for *cis*-regulatory elements. Motifs recognized by *A*. *thaliana* transcription factors involved in light signaling, such as PIFs and HY5, were found in both sequences. *SlPIF1a* promoter showed PIF and HY5 binding-motifs, PBE-box and CA-hybrid, respectively [41,42]. Additionally, CArG motifs, which are recognized by the ripening inducer transcription factor RIN [43], were also found in *SlPIF1a* promoter region. CA-hybrid and CArG motifs were also identified in *SlPIF1b* regulatory region together with the HY5-binding ACE-motif (S7A Fig). The presence of gene-specific motifs and, different number and distribution of shared motifs might, at least in part, explain the different transcriptional behavior of *SlPIF1* duplicated genes. In particular, *SlPIF1a* might be target of HY5-mediated light-induction and of the above mentioned PIF autoinhibitory mechanism. The comparison of the mRNA profiles of *SlPIF7a* and *SlPIF7b* duplicated genes was only possible in leaves because in other organs *PIF7* mRNA levels were near or below the detection threshold. *SlPIF7a* and *SlPIF7b* mRNA levels displayed opposite accumulation pattern during diel cycle, while both genes were downregulated by dark-induced senescence, *SlPIF7a* mRNA levels being 10-fold higher than those detected for *SlPIF7b* (Fig 4, Fig 5, S3 Table). In this case, the analysis of the promoter sequences showed also differential number and distribution of PIF and HY5 binding motifs: PBE-box, ACE-motif and CG-hybrid in *SlPIF7a* and; PBE-box and ACE-motif in *SlPIF7b* regulatory region (S7B Fig). To further evaluate differentially selected motifs between these pairs of duplicated genes, a promoter phylogenetic analysis was performed. Resembling the topology of the tree obtained from amino acid sequences, regulatory fragments also revealed that gene duplication predates species divergence (S8 Fig). Interestingly, none of the motifs identified in *SlPIF1s* and *SlPIF7s* is conserved between paralogs, being either *S*. *lycopersicum* exclusive or shared with *S*. *tuberosum* and *S*. *pennellii* orthologs (S9 Fig). These data reinforced that the duplicated genes have undergone functional divergence in the Solanaceae common ancestral.

According to Force et al. (1999) [44], the loss of regulatory subfunctions in the promoter region by mutation and genetic drift is the main process by which duplicated genes are preserved, as long as they retain the complete set of subfunctions from the ancestral gene. In this context, it is expected that the duplicated *loci* should complement each other and show differences at the regulatory region. This model postulates that duplicated *loci* can undergo three different fates: nonfunctionalization, neofunctionalization and subfunctionalization. The first occurs when one copy acquires disabling mutations at the promoter region, leading to gene expression loss, while the other copy remains intact. The second takes place when a copy acquires new regulatory motifs, which confers a new regulatory function to this gene. The last is caused by degenerative mutations at both *loci* leading to loss or reduction of subfunctions. Our observations suggest that *SlPIF1* genes suffered qualitative subfunctionalization, as evidenced by their opposite responsiveness to light, and neofunctionalization, since *SlPIF1a* acquired a regulatory function during fruit ripening. Whilst, *SlPIF7* duplicated *loci* appeared to have undergone quantitative subfunctionalization possibly caused by fixed reduction-of-expression mutations, which resulted in lowered expression of both copies. Moreover, it has been proposed that quantitative subfunctionalization is a transitory state to eventual neofunctionalization [45]. This seems to be the case of *SlPIF7b* gene for which, besides the reduced expression levels described above, positive selection has been also verified. A very interesting mechanism of neofunctionalization of duplicated genes in *Solanum* lineage has been recently described in tomato [46]. While in photosynthetic tissue, a CHLOROPLAST-SPECIFIC LYCOPENE β-CYCLASE (LCYβ) mediates the conversion of lycopene to β-carotene, in chromoplast, this reaction is executed by the product of the *CHROMOPLAST-SPECIFIC LYCOPENE β-CYCLASE* gene (*CYCβ*), a *LCYβ* paralog. Sequence analysis of *CYCβ* gene from a repository of tomato and wild relative accessions showed that *CYCβ* undergoes purifying selection in tomato clade. However, the abundant and diverse variations in the promoter region are likely related to regulatory neofunctionalization that played a key role in fruit color development in tomato.

The data presented here bring evidences that *SlPIF* duplicated genes (*e*.*g*. *SlPIF1a* and *SlPIF1b*), originated during *Solanum* lineage specific whole-genome triplication, have undergone sub- and neofunctionalization most likely due to variations in promoter region than in the coding region, disclosing the impact of polyploidization events during the evolution of *PIF* gene subfamily.

## Conclusions

*Solanum lycopersicum* genome harbors eight *PIF* encoding *loci*, *SlPIF1a*, *SlPIF1b*, *SlPIF3*, *SlPIF4*, *SlPIF7a*, *SlPIF7b*, *SlPIF8a* and *SlPIF8b*. *SlPIF1*, *SlPIF7* and *SlPIF8* duplications occurred during the *Solanum* lineage polyploidization event 71 (± 19.4) MYA, prior to the divergence between tomato and potato species. Transcriptional profiling revealed coincident expression patterns between tomato *SlPIF4* and Arabidopsis *AtPIF4* and *AtPIF5*, highlighting the evolutionary conserved function of PIF4 clade. Combined evolutionary analysis and transcriptional profile data indicated that *SlPIF7a* and *SlPIF7b* may have suffered quantitative subfunctionalization that reduced their expression level, followed by neofuctionalization process, supported by the differential pattern of light responsive motifs and the positive selection signatures observed. Finally, *SlPIF1a* and *SlPIF1b* promoter regions showed differential pattern of light and fruit ripening transcriptional factor binding motifs, providing also evidences for regulatory sub- and neofunctionalization. In summary, our data underlined the importance of polyploidization events on *PIF* subfamily diversification.

## Methods

### Phylogenetic, Gene Divergence Time and Evolutionary Analyses

The amino acid sequences of the five *A*. *thaliana* canonical PIF proteins (S1 Table) were used as queries to perform a BLAST search against Viridiplantae in Phytozome [47], DNA Data Bank in Japan [48], Dendrome [49], SustainPine [50], Sol Genomics [25] databases. 119 sequences with complete bHLH domain from 16 species, representing liverworts, mosses, lycophytes, gymnosperms and flowering plants, were retrieved. T-Coffee Structural-Alignment algorithm [51] was used to perform an alignment of gymnosperms and flowering plants sequences. The phylogenetic reconstruction was performed by maximum-likelihood method using JTT substitution model and validated by approximate Likelihood Ratio Test (aLRT) with the Shimodaira-Hasegawa-like (SH-like) and 100 bootstrap replicates procedures available at PhyML Interface [52].

Gene divergence time was estimated using *T* = *dS*/2*K* equation, where *T* is the divergence time, *dS* is the pairwise synonymous distance calculated in the MEGA 6 software using the corrected Nei-Gojobori method (Jukes-Cantor) [53] and, *K* is the mean substitution rate estimated for 27 *loci* belonging to three different chromosomes of *S*. *pennellii* and *S*. *lycopersicum* ([28]; 4.38x10^-9^ substitutions per site per year).

Evolutionary analysis was conducted individually for *PIF1a*, *PIF1b*, *PIF3*, *PIF4*, *PIF7a*, *PIF7b* and *PIF8a* genes using the sequences of *S*. *lycopersicum*, *S*. *pennellii* and *S*. *tuberosum*. Non-synonymous (*dN*) and synonymous (*dS*) distances and their SE values were estimated with MEGA 6. In order to preserve the reading frames, the alignment gaps were deleted prior to estimation of *dS* and *dN*. Codon bias was determined by the effective number of codons (Nc) value computed in the CodonW program [54]. Nc varies between 21 for maximum codon bias, when only one codon is used per amino acid, and 61 for minimum codon bias, when synonymous codons for each amino acid are used at similar frequencies. Three evolutionary models were evaluated using the Codeml program implemented in the PAML4.8a package and the graphical interface PAMLX 1.3.1 [55]. Phylogenetic trees were constructed using manually adjusted alignments of the coding sequences and neighbor-joining method with the optimal model of nucleotide substitution estimated by “Find Best DNA/Protein Model” using MEGA 6 software. To test for neutral evolution, the nearly neutral model (M1a) was compared with the null hypothesis, one ratio model (M0). To test positive selection, the model M2a was compared with M1a. The M0 model assumes that all codons across the sequences have the same level of *dN*/*dS*. The model M1a proposes that there two classes of codon, some with 0 ≤ *dN*/*dS* < 1 and the remainder with *dN*/*dS* = 1. Finally, model M2a divides codons into three classes: those with 0 ≤ *dN*/*dS* < 1, *dN*/*dS* = 1, and *dN*/*dS* > 1. The fit of model M1a versus M0 or M2a versus M1a is evaluated by a likelihood ratio test (LRT) comparing twice the difference in log likelihoods with a χ^2^ distribution [56]. In M1a versus M0 and M2a versus M1a the degrees of freedom (df) are 1 and 2, respectively. Bayes empirical Bayes (BEB) analyses were performed to identify positively selected residues with a BEB posterior probability 95%.

Gene collinearity was addressed by BLASTN search against tomato genome [25] using the CDS sequences within a window of 100 Kb upstream and downstream the *SlPIF1*, *SlPIF7* and *SlPIF8* duplicated genes as queries.

### Plant Material

All the experiments were performed with *Solanum lycopersicum* (cv. Micro-Tom). For deetiolation, diel cycle and dark-induced leaf senescence experiments, tomato seeds were surface sterilized and directly sown *in vitro* as described by Lira *et al*. [57]. After 120 h pre-germination in absolute darkness, seedlings were transferred to specific treatment conditions as described below. For deetiolation experiment, seedlings were either transferred to continuous white light (~100 μmol m^-2^ s^-1^) or maintained in absolute darkness for 0, 24, 48 and 72 h, after which cotyledons and hypocotyls were separately harvested. For daily cycle experiment, plants were grown under 12 h/12 h light/dark (~300 μmol m^-2^ s^-1^) photoperiod for three weeks and the second fully expanded leaves were harvested every 4 hours, for 24 hours. For dark-induced senescence, plants were grown in the same conditions as the daily cycle experiment and subsequently the plants were transferred to darkness for 0, 1, 2, 3 and 7 days for inducing leaf senescence. The second fully expanded leaves were harvested 4 h after the beginning of the light period since this day point has been shown to exhibits the highest mRNA levels of most tomato *PIF* genes. All experiments were conducted at 25 ± 1°C.

For fruit ripening experiments, plants were grown in 1 L pots in a greenhouse under automatic irrigation, at an average mean temperature of 25 ± 2°C, 11.5 h/13 h (winter/summer) photoperiod and 250–350 μmol m^-2^ s^-1^ PAR irradiance. Fruits at MG stage were harvested about 30 days after anthesis (dpa) and were transferred to continuous white light (400 to 800nm, ~50 μmol m^-2^ s^-1^) or maintained under absolute darkness until reaching distinct ripening stages in a temperature-controlled growth chamber maintained at 25 ± 2°C and air relative humidity at 80 ± 5%. Top and bottom illumination was applied in order to homogenize the light environment surrounding the fruits. Since the beginning of the treatments, fruits were placed into a 0.5 L sealed transparent vessel and continuously flushed with ethylene-free, humidified air (approximately 1 L min^-1^) in order to avoid accumulation of ethylene inside the containers. Pericarp samples (without placenta and locule walls) were harvested at MG (displaying jelly placenta, 2 days after harvesting), BR (breaker), one day after BR (BR1), three days after BR (BR3), six days after BR (BR6), twelve days after BR (BR12) stages.

Seedling, plant and fruit tissues were harvested either under the specific light conditions used for the treatments or under dim green light (~0.01 μmol m^-2^ s^-1^), as appropriate. All samples were harvested and immediately frozen in liquid nitrogen, powdered and stored at -80°C.

### Chlorophyll and Carotenoid Measurement

Chlorophyll and carotenoid extraction and analysis were carried out as described by Lira et al. [58]. When a data set showed homoscedasticity, an ANOVA test followed by a Tukey test (*P*< 0.05) was used to compare genotypes and fruit developmental stages. In the absence of homoscedasticity, a non-parametric ANOVA test was performed by applying the Kruskal–Wallis test (*P*< 0.05).

### RNA Extraction and Quantitative Polymerase Chain Reaction (qPCR) Analysis

RNA extraction, cDNA synthesis and qPCR reactions were performed as described by Quadrana et al. [59]. The primers used for qPCR are listed in S4 Table. All reactions were performed with two technical replicates and at least three biological replicates. mRNA levels were quantified using a 7500 Real-Time PCR system (Applied Biosystem) and SYBR Green Master Mix (Applied Biosystem). Absolute fluorescence data were analyzed with LinRegPCR software [60] to obtain Ct values and to calculate primer efficiency. Expression values were normalized to the mean of two constitutively expressed genes: GAGA and CAC for seedlings [61], TIP41 and EXPRESSED for leaves and CAC and EXPRESSED for fruits [59]. A permutation test lacking sample distribution assumptions [62] was applied to detect statistical differences (*P*< 0.05) in expression levels between mutants and the control using the algorithms in the fgStatistics software package [63]. For senescence analysis, the normalized expression pattern was presented by a heat map constructed with GENE-E program [64].

### Promoter Analysis

A 2 Kb fragment of the promoter sequences of *PIF1s* and *PIF7s* were retrieved from Sol Genomics Network [25]. The presence of transcription factor binding motifs was analyzed in *S*. *lycopersicum* sequences using PlantPAN 2.0 platform [65]. The promoter regions were aligned using T-Coffee Structural-Alignment algorithm [51] and the Neighbor-Joining tree was reconstructed with 100 bootstrap replicates and p-distance implemented MEGA 6 [53].

## Supporting Information

S1 Fig Phylogenetic reconstruction of PIF protein family. Phylogenetic analysis of PIF protein subfamily in Viridiplantae performed with 112 sequences from 13 species. Accession numbers of all sequences are detailed in S1 Table. Numbers at nodes represent bootstrap/approximate likelihood-ratio test (aLRT) values.
(TIF)

S2 Fig PIF functional domains. Alignment of PIF amino acid sequences from *Arabidopsis thaliana* and *Solanum lycopersicum* showing the conserved domains [25]. (a) Active phytochrome B-binding (APB) domain. Residues highlighted in red are required for APB function in *A*. *thaliana*. (b) Basic helix-loop-helix (bHLH) DNA-binding domain. (c) Active phytochrome A-binding (APA) domain.
(TIF)

S3 Fig Expression profile of *SlPIF* genes in seedling in response to light conditions. (a) Phenotype of 4-day-old dark-grown seedlings (0D) and after 24, 48 and 72 h maintained in constant light (24L, 48L and 72L) or dark (24D, 48D and 72D) conditions. Bars: 1 cm. (b) Chlorophyll content in cotyledons and hypocotyls. Different letters indicate significant differences (*P*<0.05) within treatments. (c) *SlPIF* expression profile in hypocotyls. Significant differences (*P*<0.05) among treatments are indicated by asterisks. Values shown are means ± SE of at least three biological replicates. ND: not detected.
(TIF)

S4 Fig Chlorophyll degradation and expression profile of senescence-related genes during dark-induced senescence. 3-week-old plants grown under 12 h/12 h light/dark photoperiod were transferred to constant darkness during 7 days and the second fully expanded leaves was sampled every day 4 h after the beginning of the light period. (a) Chlorophyll content along dark treatment. Significant differences (*P*<0.05) among treatments are indicated by asterisks. (b) Expression profile of *GOLDEN 2-LIKE 1* (*SlGLK1*, involved in chloroplast development, [65]), *SENESCENCE-ASSOCIATED GENE 12* (*SlSAG12*, late senescence marker, [32]), *PHEOPHYTINASE* (*SlPPH*, involved in leaf chlorophyll degradation, [56]) and, three genes tomato genes homologs to the *Arabidopsis thaliana ORESARA 1* (*SlORE1S02*, *SlORE1S03* and *SlORE1S06*, senescence-related transcription factor). Heatmap representation of the relative mRNA abundance compared to day 0. Different letters indicate statistical differences (*P*<0.05) among sampling times. Values shown are means ± SE of at least three biological replicates.
(TIF)

S5 Fig Off-vine treated fruits undergo normal ripening process. Total Chlorophyll (a) and total carotenoid (b) levels were measured spectrophotometrically. Fruits were harvested at MG (mature-green) stage and left to ripen under constant light or dark conditions. Pericarp samples were harvested at MG (two days after the beginning of treatment), BR (breaker), BR1 (1 day after BR), BR3, BR6 and BR12 stages. Asterisks and letters represent significant (P<0.05) differences between treatments and stages, respectively. Values shown are means ± SE of at least three biological replicates.
(TIF)

S6 Fig Microsynteny along the genomic regions flanking duplicated genes. Gene collinearity was addressed within a window of 100 Kb upstream and downstream the *SlPIF1* (a), *SlPIF7* (b) and *SlPIF8* (c) duplicated genes. *SlPIF1b* (Solyc06g008030), *SlPIF1a* (Solyc09g063010), *SlPIF7a* (Solyc03g115540), *SlPIF1b* (Solyc06g069600), *SlPIF8a* (Solyc01g090790) and *SlPIF8b* (Solyc10g018510) are highlighted in red. Collinear *loci* are indicated by arrows. The number of predicted genes within the intervals are indicated between parentheses.
(TIF)

S7 Fig Motifs identified in *SlPIF* gene promoter region. Fragments of 2 kb upstream the translation initiation site of *SlPIF1a* and *SlPIF1b* (a) and, *SlPIF7a* and *SlPIF7b* (b) genes are represented by a blue line. Motif positions are indicated by triangles. CArG [42], PBE-box [40], CA-hybrid, CG-hybrid and ACE-motif [41].
(TIF)

S8 Fig Phylogenetic analysis of duplicated gene promoter sequences. (TIF)

S9 Fig Motif conservation in duplicated gene promoter sequences. The motifs identified in S7 Fig are highlighted in yellow (CArG [42]), blue (PBE-box [40]), green (CA-hybrid [41]), orange (CG-hybrid [41]) and pink (ACE-motif [41]).
(PDF)

S1 Table Sequences used in phylogenetic reconstruction of PIF protein subfamily. (XLSX)

S2 Table Percentage of identity between *Arabidopsis thaliana* and *Solanum lycopersicum* homologs. (XLSX)

S3 Table Relative expression of *SlPIF* genes in the tested organs. (XLSX)

S4 Table Primers used for qPCR analyses. (XLSX)

S1 Text Fasta alignment used for phylogenetic analysis. (FAS)

## References

[1] Leivar P, Quail PH. PIFs: Pivotal components in a cellular signaling hub. Trends Plant Sci. 2011;16: 19–28. 10.1016/j.tplants.2010.08.003 20833098 PMC3019249

[2] Bino RJ, De Vos CHR, Lieberman M, Hall RD, Bovy A, Jonker HH, et al The light-hyperresponsive high pigment-2^dg^ mutation of tomato: Alterations in the fruit metabolome. New Phytol. 2005;166: 427–438. 10.1111/j.1469-8137.2005.01362.x 15819907

[3] Azari R, Tadmor Y, Meir A, Reuveni M, Evenor D, Nahon S, et al Light signaling genes and their manipulation towards modulation of phytonutrient content in tomato fruits. Biotechnol Adv. 2010;28: 108–118. 10.1016/j.biotechadv.2009.10.003 19850117

[4] Liu Y, Roof S, Ye Z, Barry C, Van Tuinent A, Vrebalov J, et al Manipulation of light signal transduction as a means of modifying fruit nutritional quality in tomato. Proc Natl Acad Sci U S A. 2004;101: 9897–9902. 10.1073/pnas.0400935101 15178762 PMC470770

[5] The Tomato Genome Consortium. The tomato genome sequence provides insights into fleshy fruit evolution. Nature. 2012;485: 635–641. 10.1038/nature11119 22660326 PMC3378239

[6] Rensing SA. Gene duplication as a driver of plant morphogenetic evolution. Curr Opin Plant Biol. Elsevier Ltd; 2014;17: 43–48. 10.1016/j.pbi.2013.11.002 24507493

[7] Hauser B, Cordonnier-Pratt MM, Daniel-Vedele F, Pratt LH. The phytochrome gene family in tomato includes a novel subfamily. Plant Mol Biol. 1995;29: 1143–1155. 10.1007/BF00020458 8616214

[8] Weller JL, Schreuder MEL, Smith H, Koornneef M, Kendrick RE. Physiological interactions of phytochromes A, B1 and B2 in the control of development in tomato. Plant J. 2000;24: 345–356. 10.1046/j.1365-313X.2000.00879.x 11069708

[9] Husaineid SSH, Kok RA, Schreuder MEL, Hanumappa M, Cordonnier-Pratt MM, Pratt LH, et al Overexpression of homologous phytochrome genes in tomato: Exploring the limits in photoperception. J Exp Bot. 2007;58: 615–626. 10.1093/jxb/erl253 17251177

[10] Gupta SK, Sharma S, Santisree P, Kilambi HV, Appenroth K, Sreelakshmi Y, et al Complex and shifting interactions of phytochromes regulate fruit development in tomato. Plant, Cell Environ. 2014;37: 1688–1702. 10.1111/pce.12279 24433205

[11] Cordeiro AM, Figueiredo DD, Tepperman J, Borba AR, Lourenço T, Abreu IA, et al Rice phytochrome-interacting factor protein OsPIF14 represses OsDREB1B gene expression through an extended N-box and interacts preferentially with the active form of phytochrome B. Biochim Biophys Acta—Gene Regul Mech. 2016;1859: 393–404. 10.1016/j.bbagrm.2015.12.008 26732823 PMC4824199

[12] Llorente B, D’Andrea L, Ruiz-Sola MA, Botterweg E, Pulido P, Andilla J, et al Tomato fruit carotenoid biosynthesis is adjusted to actual ripening progression by a light-dependent mechanism. Plant J. 2015; 10.1111/tpj.13094 26648446

[13] Inoue K, Nishihama R, Kataoka H, Hosaka M, Manabe R, Nomoto M, et al Phytochrome signaling is mediated by PHYTOCHROME INTERACTING FACTOR in the liverwort Marchantia polymorpha. Plant Cell. 2016;28: tpc.01063.2015. 10.1105/tpc.15.01063 27252292 PMC4944405

[14] Kumar I, Swaminathan K, Hudson K, Hudson ME. Evolutionary divergence of phytochrome protein function in *Zea mays* PIF3 signaling. J Exp Bot. 2016;67: 4231–4240. 10.1093/jxb/erw217 27262126 PMC5301934

[15] Monte E, Tepperman JM, Al-Sady B, Kaczorowski KA, Alonso JM, Ecker JR, et al The phytochrome-interacting transcription factor, PIF3, acts early, selectively, and positively in light-induced chloroplast development. Proc Natl Acad Sci U S A. 2004;101: 16091–8. 10.1073/pnas.0407107101 15505214 PMC528976

[16] Song Y, Yang C, Gao S, Zhang W, Li L, Kuai B. Age-Triggered and Dark-Induced Leaf Senescence Require the bHLH Transcription Factors PIF3, 4, and 5. Mol Plant. 2014;7: 1776–1787. 10.1093/mp/ssu109 25296857 PMC4261840

[17] Sakuraba Y, Jeong J, Kang M-Y, Kim J, Paek N-C, Choi G. Phytochrome-interacting transcription factors PIF4 and PIF5 induce leaf senescence in Arabidopsis. Nat Commun. 2014;5: 4636 10.1038/ncomms5636 25119965

[18] Zhang Y, Liu Z, Chen Y, He JX, Bi Y. PHYTOCHROME-INTERACTING FACTOR 5 (PIF5) positively regulates dark-induced senescence and chlorophyll degradation in Arabidopsis. Plant Sci. 2015;237: 57–68. 10.1016/j.plantsci.2015.05.010 26089152

[19] Moon J, Zhu L, Shen H, Huq E. PIF1 directly and indirectly regulates chlorophyll biosynthesis to optimize the greening process in Arabidopsis. Proc Natl Acad Sci U S A. 2008;105: 9433–9438. 10.1073/pnas.0803611105 18591656 PMC2453699

[20] Toledo-Ortiz G, Huq E, Rodríguez-Concepción M. Direct regulation of phytoene synthase gene expression and carotenoid biosynthesis by phytochrome-interacting factors. Proc Natl Acad Sci U S A. 2010;107: 11626–11631. 10.1073/pnas.0914428107 20534526 PMC2895139

[21] Llorente B, D’Andrea L, Rodriguez-Concepción M. Evolutionary recycling of light signaling components in fleshy fruits: new insights on the role of pigments to monitor ripening. Front. Plant Sci. 2016; 10.3389/fpls.2016.00263 27014289 PMC4780243

[22] Pires N, Dolan L. Early evolution of bHLH proteins in plants. Plant Signal Behav. 2010;5: 911–912. doi: 10.1093/molbev/msp288 20523129 PMC3115042

[23] Toledo-Ortiz G, Huq E, Quail PH. The Arabidopsis Basic / Helix-Loop-Helix Transcription Factor Family. Plant Cell. 2003;15: 1749–1770. 10.1105/tpc.013839.et 12897250 PMC167167

[24] Groszmann M, Paicu T, Alvarez JP, Swain SM, Smyth DR. SPATULA and ALCATRAZ, are partially redundant, functionally diverging bHLH genes required for Arabidopsis gynoecium and fruit development. Plant J. 2011;68: 816–829. 10.1111/j.1365-313X.2011.04732.x 21801252

[25] Sol Genomics Network [Internet]. 2014. Available: https://solgenomics.net/.

[26] Khanna R, Huq E, Kikis E a, Al-Sady B, Lanzatella C, Quail PH. A novel molecular recognition motif Nnecessary for targeting photoactivated phytochrome signaling to specific Basic Helix-Loop-Helix transcription factors. Plant Cell. 2004;16: 3033–3044. 10.1105/tpc.104.025643.1 15486100 PMC527196

[27] Kamenetzky L, Asis R, Bassi S, de Godoy F, Bermudez L, Fernie AR, et al Genomic Analysis of Wild Tomato Introgressions Determining Metabolism- and Yield-Associated Traits. Plant Physiol. 2010;152: 1772–1786. 10.1104/pp.109.150532 20118271 PMC2850009

[28] Maddison WP. Gene trees in species trees. Syst Biol. 1997;46: 523–536. 10.1093/sysbio/46.3.523

[29] Pulquério MJF, Nichols RA. Dates from the molecular clock: how wrong can we be? Trends Ecol Evol. 2007;22: 180–184. 10.1016/j.tree.2006.11.013 17157408

[30] Wang Y, Diehl A, Wu F, Vrebalov J, Giovannoni J, Siepel A, et al Sequencing and comparative analysis of a conserved syntenic segment in the solanaceae. Genetics. 2008;180: 391–408. 10.1534/genetics.108.087981 18723883 PMC2535690

[31] Powell ALT, Nguyen C v., Hill T, Cheng kalai lam, Figueroa-Balderas R, Aktas H, et al Uniform ripening encodes a Golden 2-like transcription factor regulating tomato fruit chloroplast development. Science. 2012;336: 1711–5. 10.1126/science.1222218 22745430

[32] Guyer L, Hofstetter SS, Christ B, Lira BS, Rossi M, Hörtensteiner S. Different mechanisms are responsible for chlorophyll dephytylation during fruit ripening and leaf senescence in tomato. Plant Physiol. 2014;166: 44–56. 10.1104/pp.114.239541 25033826 PMC4149727

[33] Weaver LM, Gan S, Quirino B, Amasino RM. A comparison of the expression patterns of several senescence-associated genes in response to stress and hormone treatment. Plant Mol Biol. 1998;37: 455–469. 10.1023/A:1005934428906 9617813

[34] Leivar P, Monte E. PIFs: systems integrators in plant development. Plant Cell. 2014;26: 56–78. 10.1105/tpc.113.120857 24481072 PMC3963594

[35] Bell CD, Soltis DE, Soltis PS. The age of the angiosperms: a molecular timescale without a clock. Evolution. 2005;59: 1245–58. 10.1554/05-005 16050101

[36] Wilke T, Schultheiß R, Albrecht C. As Time Goes by: A Simple Fool’s Guide to Molecular Clock Approaches in Invertebrates. Am Malacol Bull. 2009;27: 25–45. 10.4003/006.027.0203

[37] Pedmale U V., Huang SSC, Zander M, Cole BJ, Hetzel J, Ljung K, et al Cryptochromes Interact Directly with PIFs to Control Plant Growth in Limiting Blue Light. Cell. Elsevier Inc.; 2016;164: 233–245. 10.1016/j.cell.2015.12.018 26724867 PMC4721562

[38] Soy J, Leivar P, Monte E. PIF1 promotes phytochrome-regulated growth under photoperiodic conditions in Arabidopsis together with PIF3, PIF4, and PIF5. J Exp Bot. 2014;65: 2925–2936. 10.1093/jxb/ert465 24420574 PMC4056538

[39] Lee C-M, Thomashow MF. Photoperiodic regulation of the C-repeat binding factor (CBF) cold acclimation pathway and freezing tolerance in Arabidopsis thaliana. Proc Natl Acad Sci U S A. 2012;109: 15054–9. 10.1073/pnas.1211295109 22927419 PMC3443188

[40] Kidokoro S, Maruyama K, Nakashima K, Imura Y, Narusaka Y, Shinwari ZK, et al The Phytochrome-Interacting Factor PIF7 negatively regulates DREB1 expression under circadian control in Arabidopsis. Plant Physiol. 2009;151:2046–2057. 10.1104/pp.109.147033 19837816 PMC2785984

[41] Zhang X, Yao D, Wang Q, Xu W, Wei Q, Wang C, et al mRNA-seq analysis of the *Gossypium arboreum* transcriptome reveals tissue selective signaling in response to water wtress during seedling stage. PLoS One. 2013;8 10.1371/journal.pone.0054762 23382961 PMC3557298

[42] Lee J, He K, Stolc V, Lee H, Figueroa P, Gao Y, et al Analysis of Transcription Factor HY5 Genomic Binding Sites Revealed Its Hierarchical Role in Light Regulation of Development. Plant Cell Online. 2007;19: 731–749. 10.1105/tpc.106.047688 17337630 PMC1867377

[43] Fujisawa M, Shima Y, Higuchi N, Nakano T, Koyama Y, Kasumi T, et al Direct targets of the tomato-ripening regulator RIN identified by transcriptome and chromatin immunoprecipitation analyses. Planta. 2012;235: 1107–1122. 10.1007/s00425-011-1561-2 22160566

[44] Force A, Lynch M, Postlethwait J. Preservation of duplicate genes by subfunctionalization. Am Zool. 1999;39: 0. doi: 10101175

[45] Teufel AI, Liu L, Liberles DA. Models for gene duplication when dosage balance works as a transition state to subsequent neo-or sub-functionalization BMC Evol Biol. BMC Evolutionary Biology; 2016;16: 45 10.1186/s12862-016-0616-1 26897341 PMC4761171

[46] Mohan V, Gupta S, Thomas S, Mickey H, Charakana C, Chauhan VS, et al Tomato Fruits Show Wide Phenomic Diversity but Fruit Developmental Genes Show Low Genomic Diversity. PLoS One. 2016;11: e0152907 10.1371/journal.pone.0152907 27077652 PMC4831840

[47] Phytozome 11: The Plant Genomics Resource [Internet]. 2015. Available: https://phytozome.jgi.doe.gov/. Accessed 31 July 2016.

[48] DNA Data Bank of Japan [Internet]. 2016. Available: http://www.ddbj.nig.ac.jp/. Accessed 31 July 2016.

[49] Dendrome. A Forest Tree Genome Database. [Internet]. Available: http://dendrome.ucdavis.edu. Accessed 31 July 2016.

[50] SustainPine [Internet]. Available: http://www.scbi.uma.es/sustainpinedb/home_page. Accessed 31 July 2016.

[51] Notredame C., Higgins D. G., & Heringa J, Notredame C, Higgins DG, Heringa J. T-coffee: a novel method for fast and accurate multiple sequence alignment. J Mol Biol. 2000;302: 205–217. 10.1006/jmbi.2000.4042 10964570

[52] HIV Sequence Database. PhyML interface. [Internet]. Available: http://www.hiv.lanl.gov/content/sequence/PHYML/interface.html. Accessed 31 July 2016.

[53] Tamura K, Stecher G, Peterson D, Filipski A, Kumar S. MEGA6: Molecular evolutionary genetics analysis version 6.0. Mol Biol Evol. 2013;30: 2725–2729. 10.1093/molbev/mst197 24132122 PMC3840312

[54] Mobile @Pasteur [Internet]. 2011. Available: http://mobyle.pasteur.fr/cgi-bin/portal.py?-form1⁄4codonw. Accessed 31 July 2016.

[55] PAML-X: A GUI for PAML [Internet]. Available: http://abacus.gene.ucl.ac.uk/software/paml.html#PAMLx. Accessed 31 July 2016.

[56] Yang Z. PAML 4: Phylogenetic analysis by maximum likelihood. Mol Biol Evol. 2007;24: 1586–1591. 10.1093/molbev/msm088 17483113

[57] Lira BS, Setta N, Rosado D, Almeida J, Freschi L, Rossi M. Plant degreening: evolution and expression of tomato (*Solanum lycopersicum*) dephytylation enzymes. Gene. 2014;546: 359–66. 10.1016/j.gene.2014.05.051 24865932

[58] Lira BS, Rosado D, Almeida J, De Souza AP, Buckeridge MS, Purgatto E, et al Pheophytinase knockdown impacts carbon metabolism and nutraceutical content under normal growth conditions in tomato. Plant Cell Physiol. 2016;57: 642–653. 10.1093/pcp/pcw021 26880818

[59] Quadrana L, Almeida J, Otaiza SN, Duffy T, da Silva JVC, de Godoy F, et al Transcriptional regulation of tocopherol biosynthesis in tomato. Plant Mol Biol. 2013;81: 309–325. 10.1007/s11103-012-0001-4 23247837

[60] Ruijter JM, Ramakers C, Hoogaars WMH, Karlen Y, Bakker O, van den hoff MJB, et al Amplification efficiency: Linking baseline and bias in the analysis of quantitative PCR data. Nucleic Acids Res. 2009;37 10.1093/nar/gkp045 19237396 PMC2665230

[61] Melo NKG, Bianchetti RE, Lira BS, Oliveira PMR, Zuccarelli R, Dias DLO, et al Nitric Oxide, Ethylene, and Auxin Cross Talk Mediates Greening and Plastid Development in Deetiolating Tomato Seedlings. Plant Physiol. 2016;170: 2278–2294. 10.1104/pp.16.00023 26829981 PMC4825133

[62] Pfaffl MW, Horgan GW, Dempfle L. Relative expression software tool (REST) for group-wise comparison and statistical analysis of relative expression results in real-time PCR. Nucleic Acids Res. 2002;30: e36 10.1093/nar/30.9.e36 11972351 PMC113859

[63] Di Rienzo J. fgStatistics Statistical software for the analysis of experiments of functional genomics. [Internet]. RDNDA, Argentina.; 2012 Available: http://sites.google.com/site/fgStatistics/.

[64] GENE-E [Internet]. Available: http://www.broadinstitute.org/cancer/software/GENE-E/. Accessed 31 July 2016.

[65] Chow C-N, Zheng H-Q, Wu N-Y, Chien C-H, Huang H-D, Lee T-Y, et al PlantPAN 2.0: an update of plant promoter analysis navigator for reconstructing transcriptional regulatory networks in plants. Nucleic Acids Res. 2015;44: D1154–60. 10.1093/nar/gkv1035 26476450 PMC4702776

