# Supplementary figures and images for: Phytochrome Interacting Factors (PIFs) in Solanum lycopersicum: Diversity, Evolutionary History and Expression Profiling during Different Developmental Processes
Source: PLoS One. 2016 Nov 1;11(11):e0165929. doi: 10.1371/journal.pone.0165929 (PMC5089782; doi:10.1371/journal.pone.0165929)

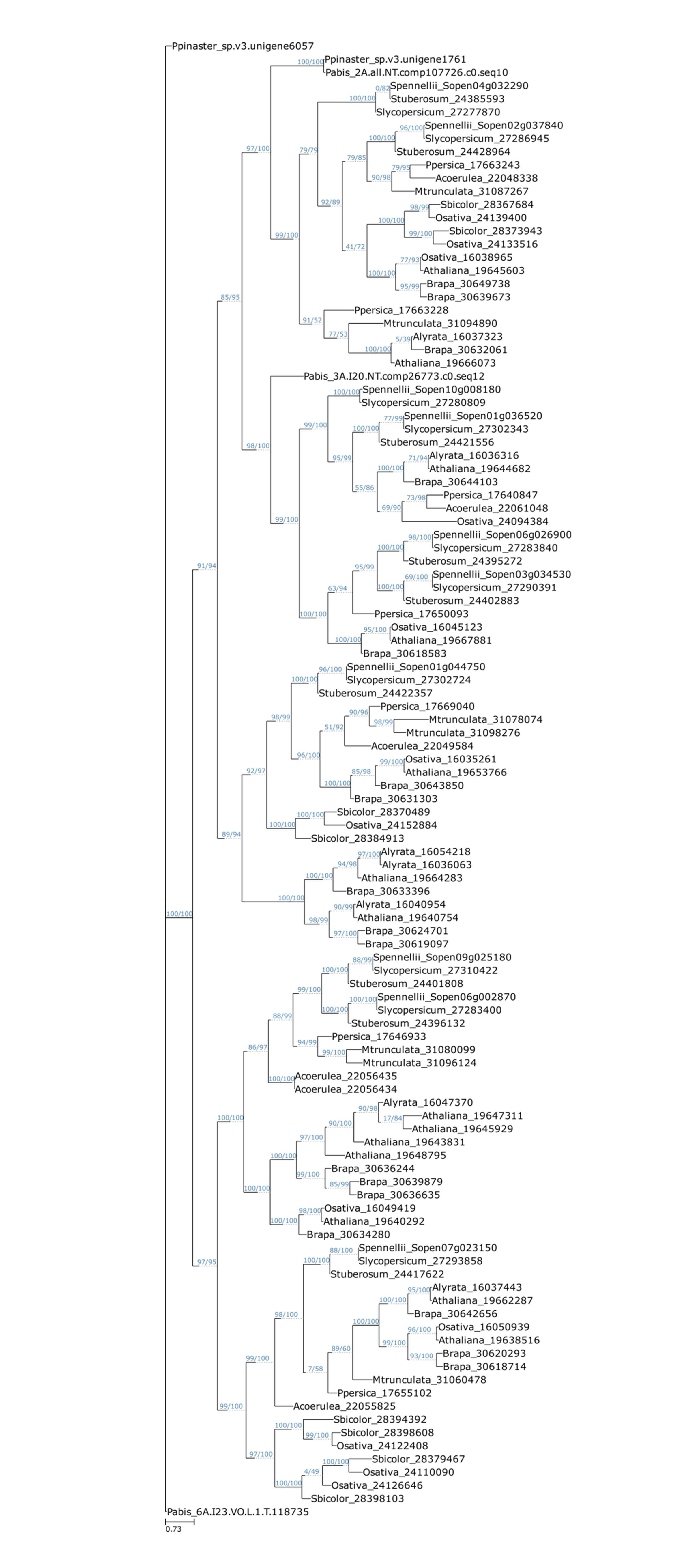

Supplement: S1 Fig — Phylogenetic analysis of PIF protein subfamily in Viridiplantae performed with 112 sequences from 13 species. Accession numbers of all sequences are detailed in S1 Table. Numbers at nodes represent bootstrap/approximate likelihood-ratio test (aLRT) values. (TIF) [file pone.0165929.s001.tif]

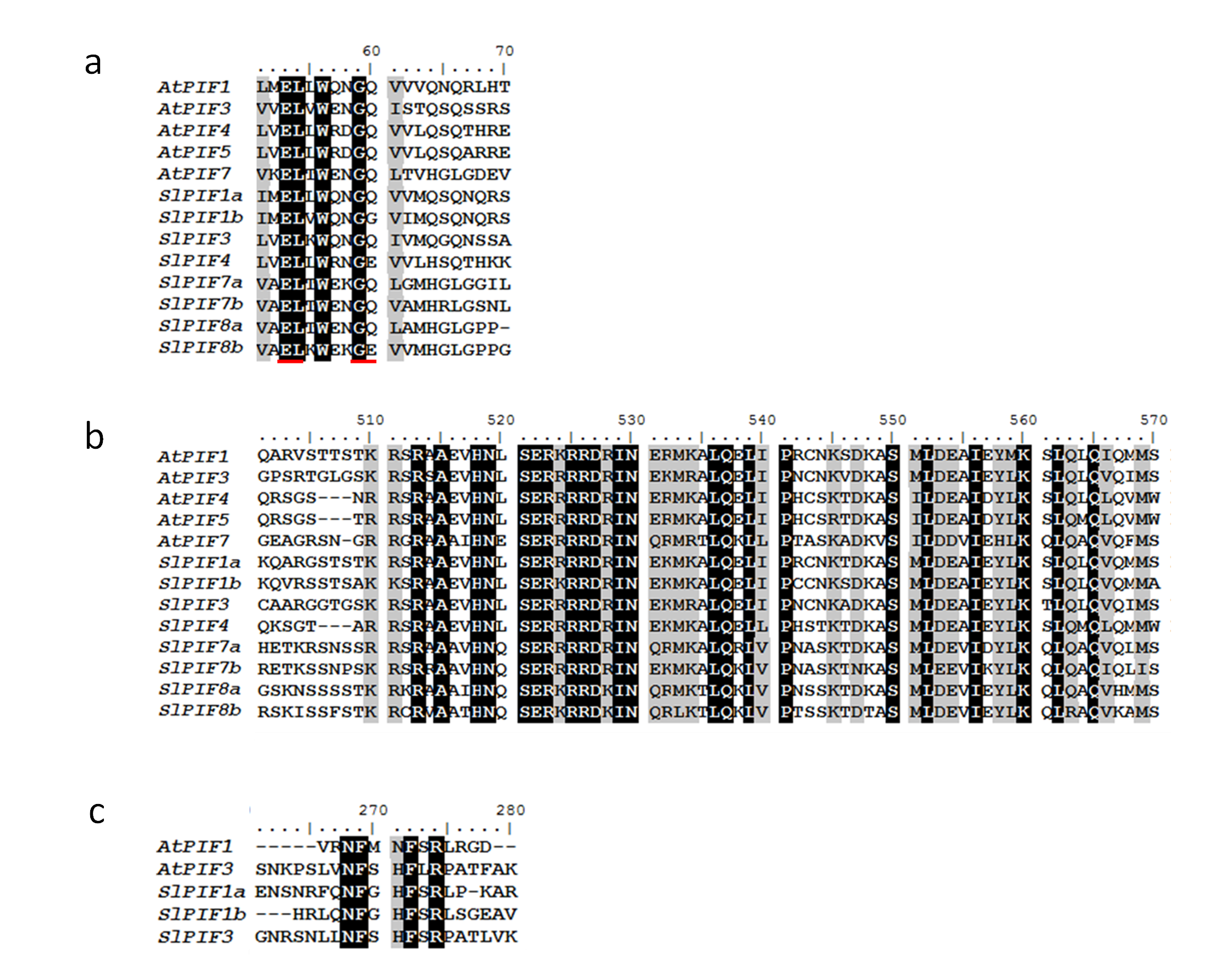

Supplement: S2 Fig — Alignment of PIF amino acid sequences from Arabidopsis thaliana and Solanum lycopersicum showing the conserved domains [25]. (a) Active phytochrome B-binding (APB) domain. Residues highlighted in red are required for APB function in A. thaliana. (b) Basic helix-loop-helix (bHLH) DNA-binding domain. (c) Active phytochrome A-binding (APA) domain. (TIF) [file pone.0165929.s002.tif]

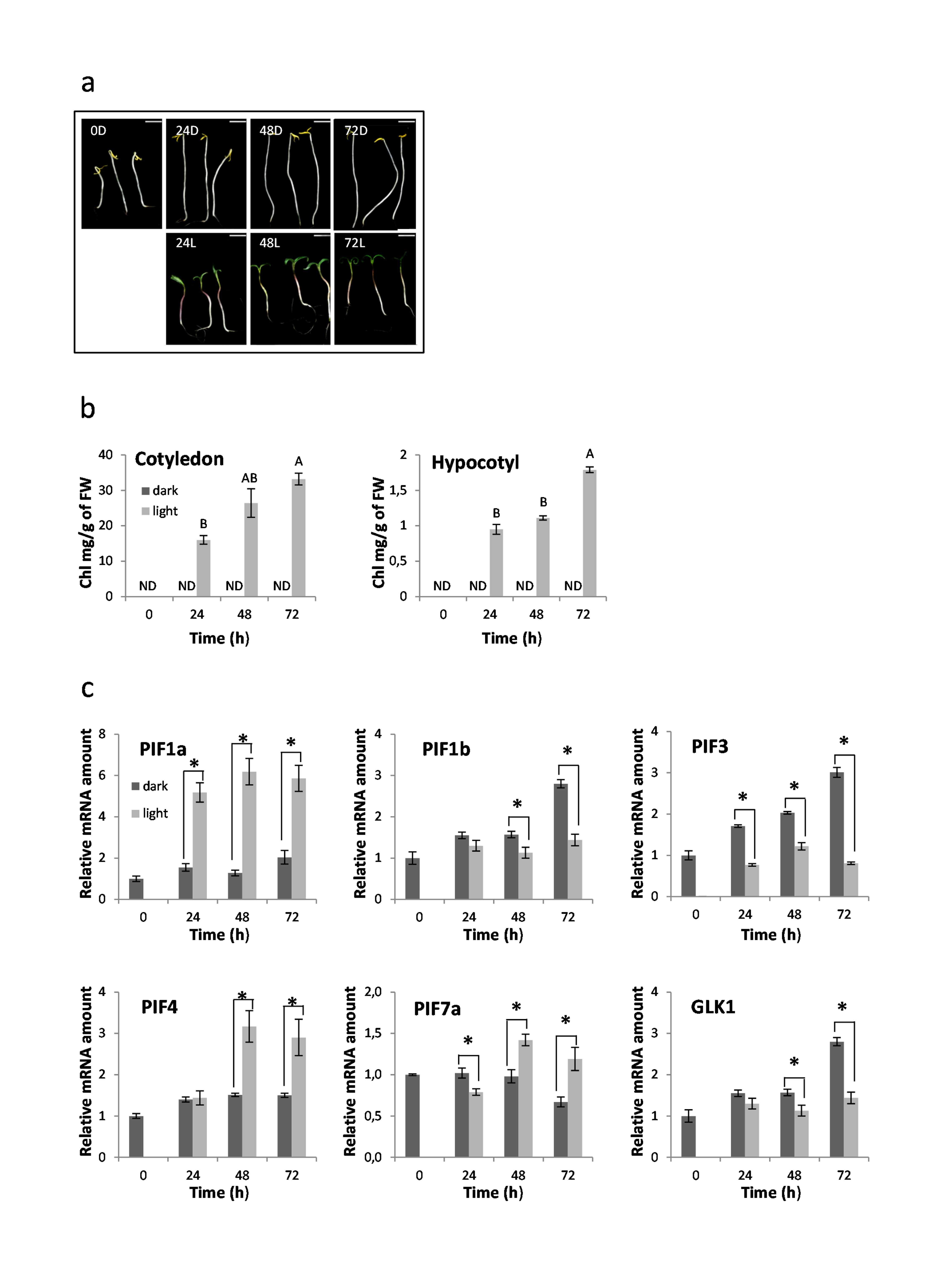

Supplement: S3 Fig — (a) Phenotype of 4-day-old dark-grown seedlings (0D) and after 24, 48 and 72 h maintained in constant light (24L, 48L and 72L) or dark (24D, 48D and 72D) conditions. Bars: 1 cm. (b) Chlorophyll content in cotyledons and hypocotyls. Different letters indicate significant differences (P<0.05) within treatments. (c) SlPIF expression profile in hypocotyls. Significant differences (P<0.05) among treatments are indicated by asterisks. Values shown are means ± SE of at least three biological replicates. ND: not detected. (TIF) [file pone.0165929.s003.tif]

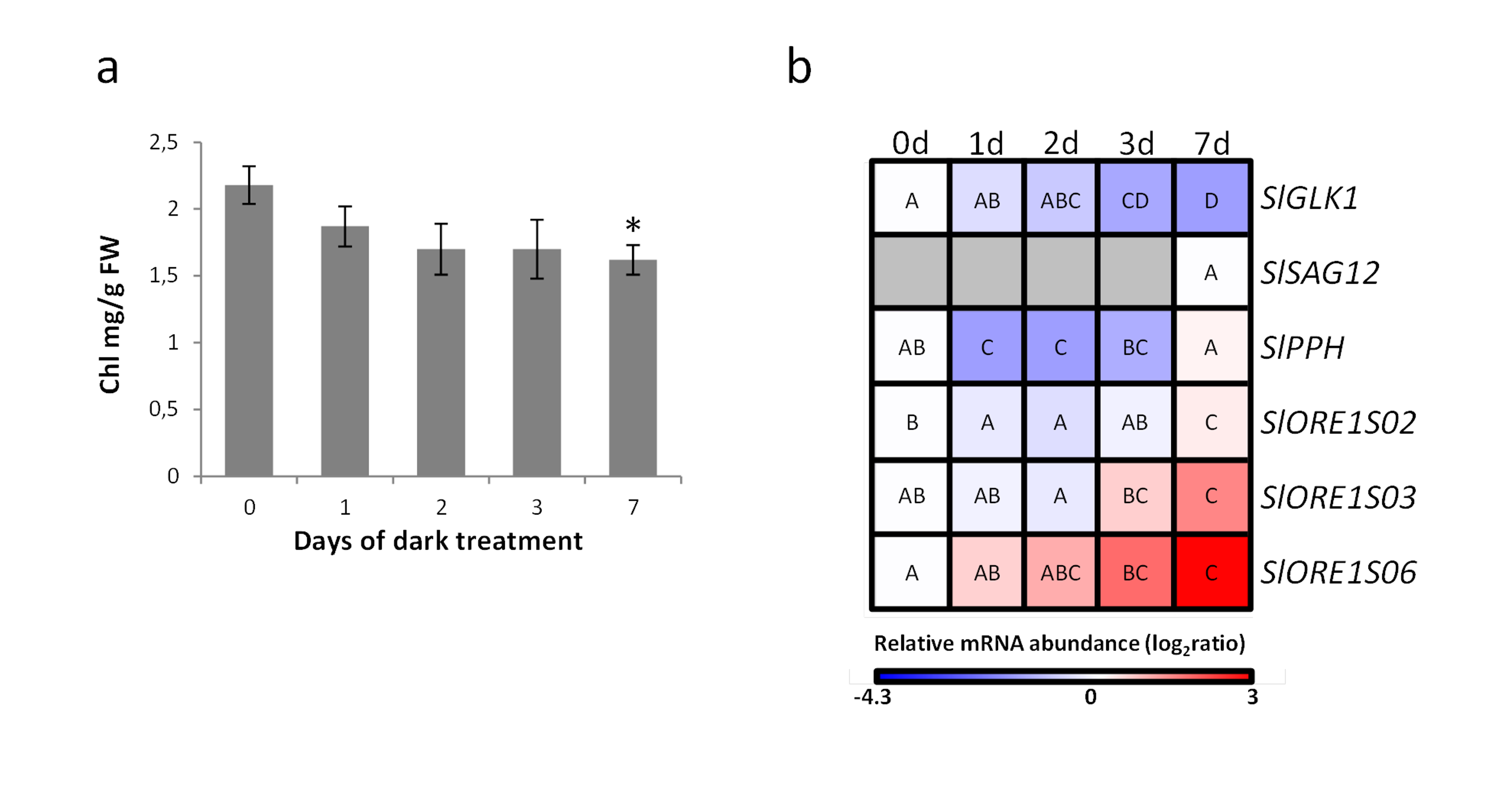

Supplement: S4 Fig — 3-week-old plants grown under 12 h/12 h light/dark photoperiod were transferred to constant darkness during 7 days and the second fully expanded leaves was sampled every day 4 h after the beginning of the light period. (a) Chlorophyll content along dark treatment. Significant differences (P<0.05) among treatments are indicated by asterisks. (b) Expression profile of GOLDEN 2-LIKE 1 (SlGLK1, involved in chloroplast development, [65]), SENESCENCE-ASSOCIATED GENE 12 (SlSAG12, late senescence marker, [32]), PHEOPHYTINASE (SlPPH, involved in leaf chlorophyll degradation, [56]) and, three genes tomato genes homologs to the Arabidopsis thaliana ORESARA 1 (SlORE1S02, SlORE1S03 and SlORE1S06, senescence-related transcription factor). Heatmap representation of the relative mRNA abundance compared to day 0. Different letters indicate statistical differences (P<0.05) among sampling times. Values shown are means ± SE of at least three biological replicates. (TIF) [file pone.0165929.s004.tif]

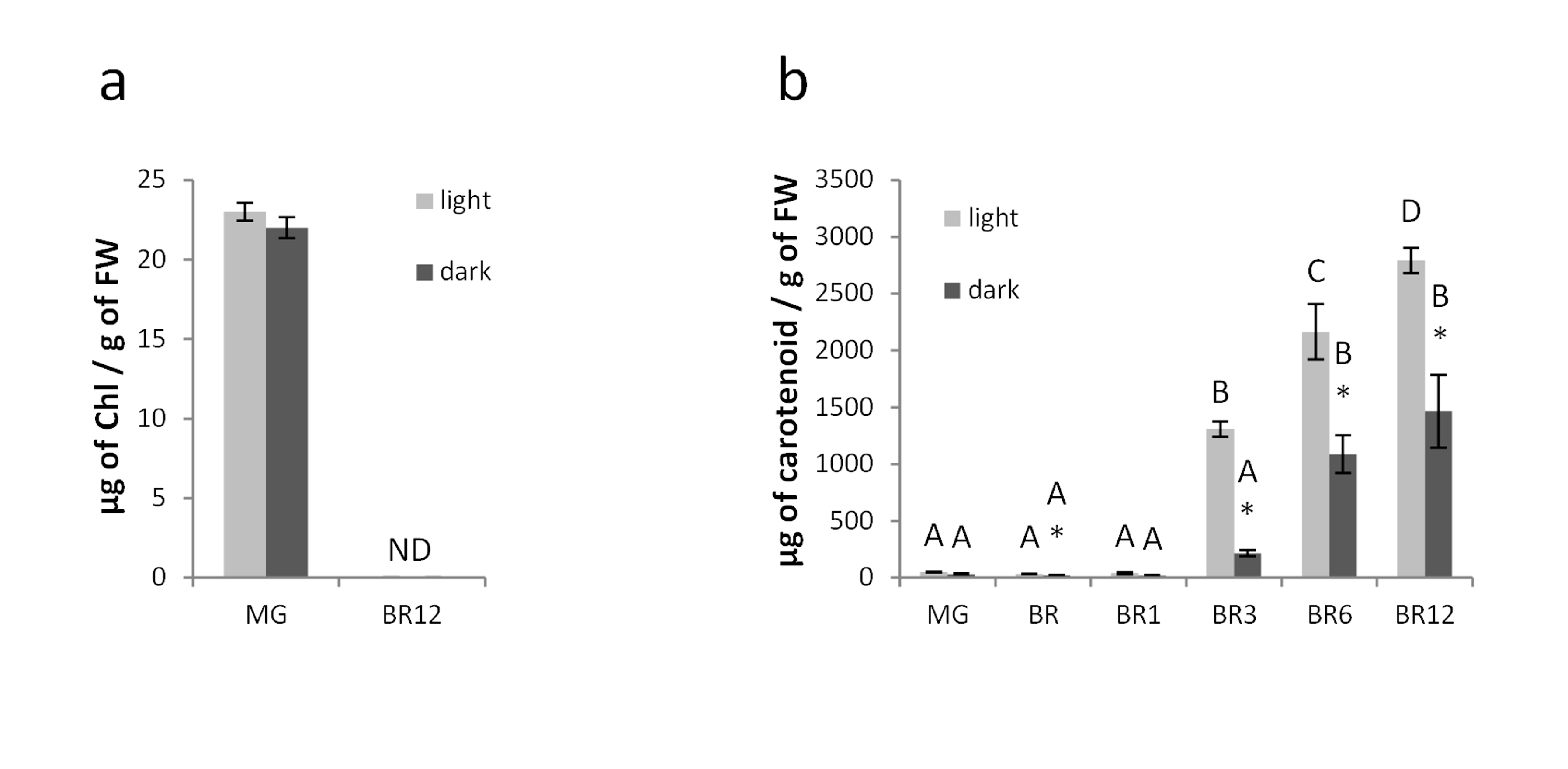

Supplement: S5 Fig — Total Chlorophyll (a) and total carotenoid (b) levels were measured spectrophotometrically. Fruits were harvested at MG (mature-green) stage and left to ripen under constant light or dark conditions. Pericarp samples were harvested at MG (two days after the beginning of treatment), BR (breaker), BR1 (1 day after BR), BR3, BR6 and BR12 stages. Asterisks and letters represent significant (P<0.05) differences between treatments and stages, respectively. Values shown are means ± SE of at least three biological replicates. (TIF) [file pone.0165929.s005.tif]

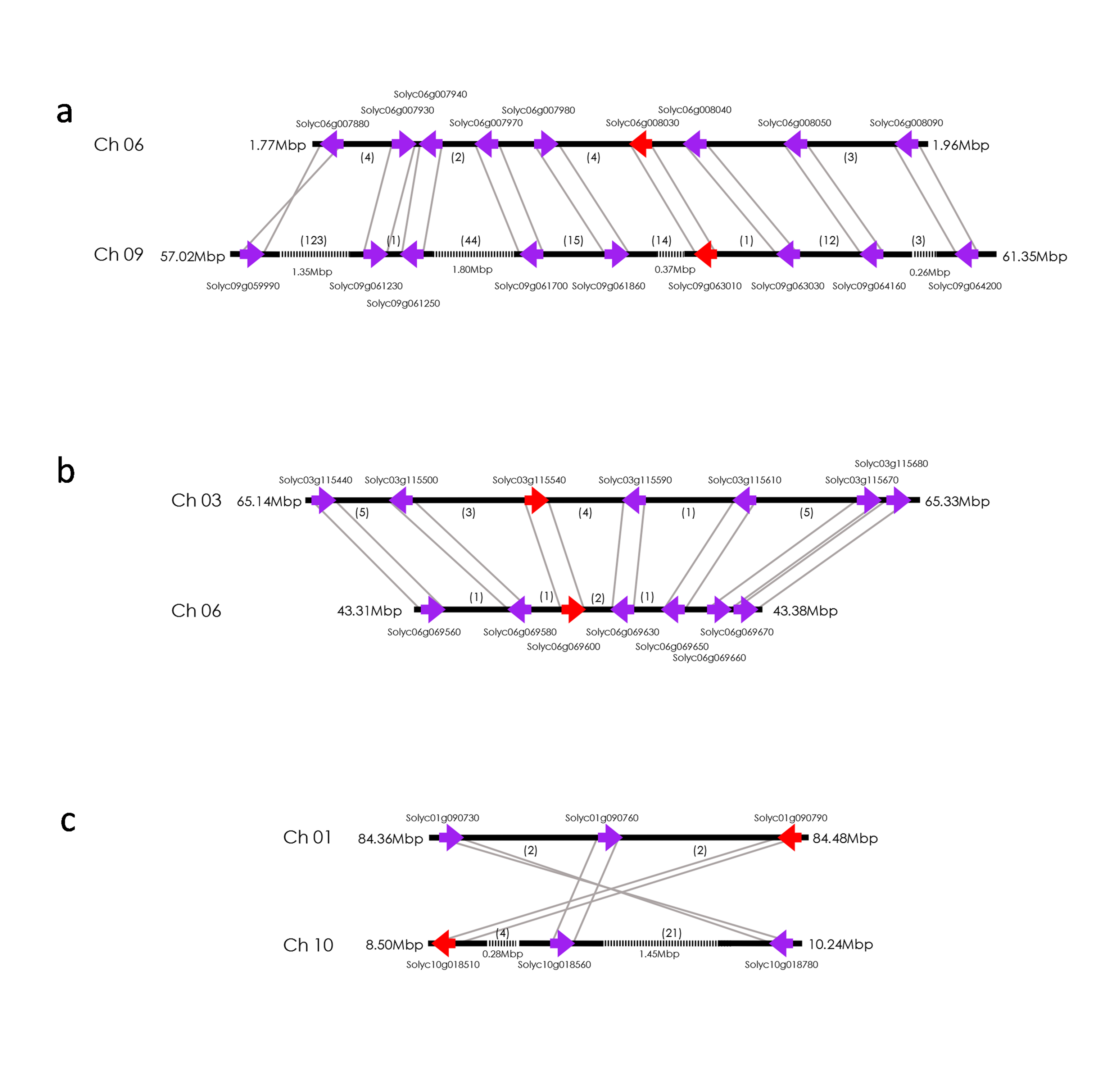

Supplement: S6 Fig — Gene collinearity was addressed within a window of 100 Kb upstream and downstream the SlPIF1 (a), SlPIF7 (b) and SlPIF8 (c) duplicated genes. SlPIF1b (Solyc06g008030), SlPIF1a (Solyc09g063010), SlPIF7a (Solyc03g115540), SlPIF1b (Solyc06g069600), SlPIF8a (Solyc01g090790) and SlPIF8b (Solyc10g018510) are highlighted in red. Collinear loci are indicated by arrows. The number of predicted genes within the intervals are indicated between parentheses. (TIF) [file pone.0165929.s006.tif]

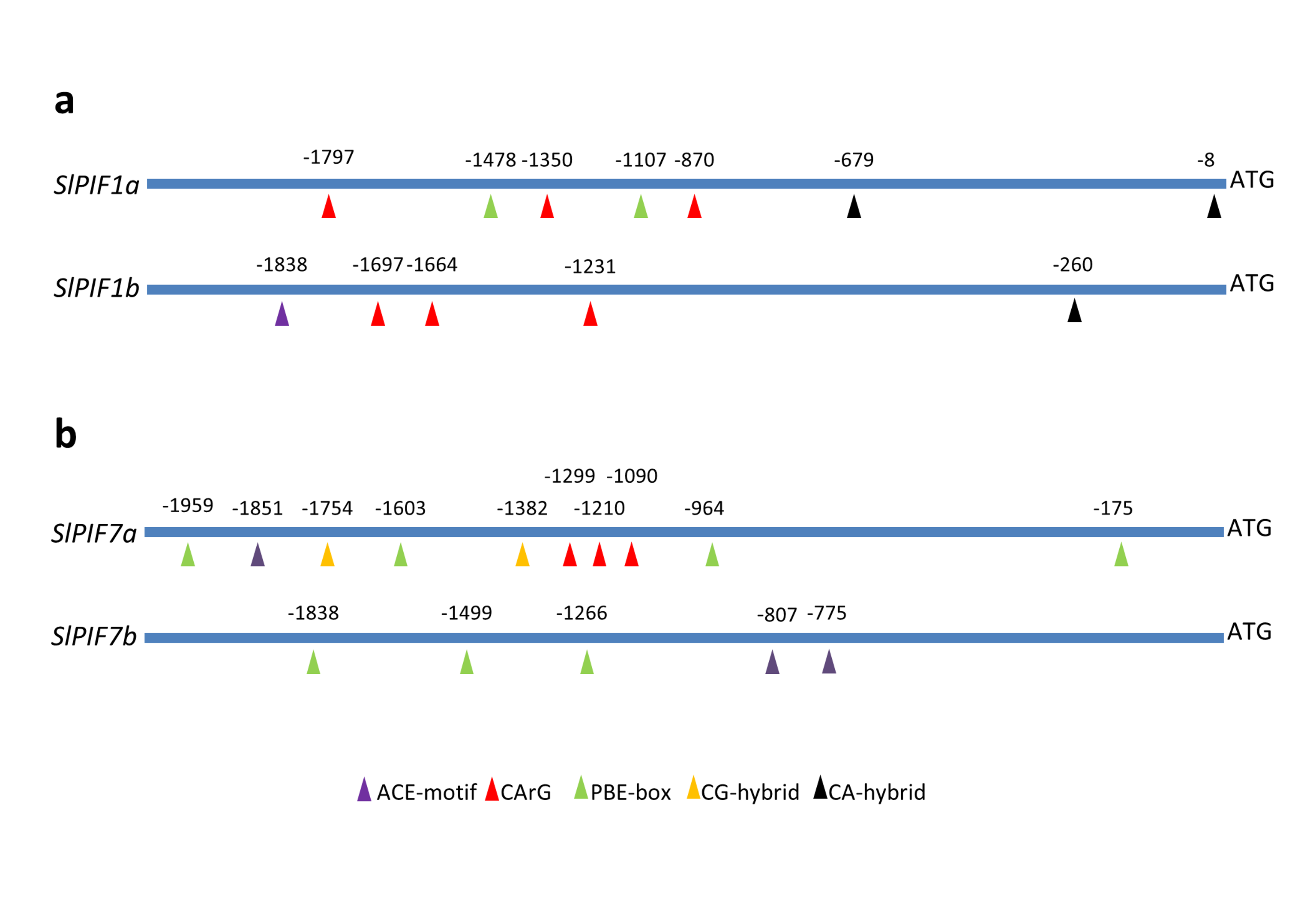

Supplement: S7 Fig — Fragments of 2 kb upstream the translation initiation site of SlPIF1a and SlPIF1b (a) and, SlPIF7a and SlPIF7b (b) genes are represented by a blue line. Motif positions are indicated by triangles. CArG [42], PBE-box [40], CA-hybrid, CG-hybrid and ACE-motif [41]. (TIF) [file pone.0165929.s007.tif]

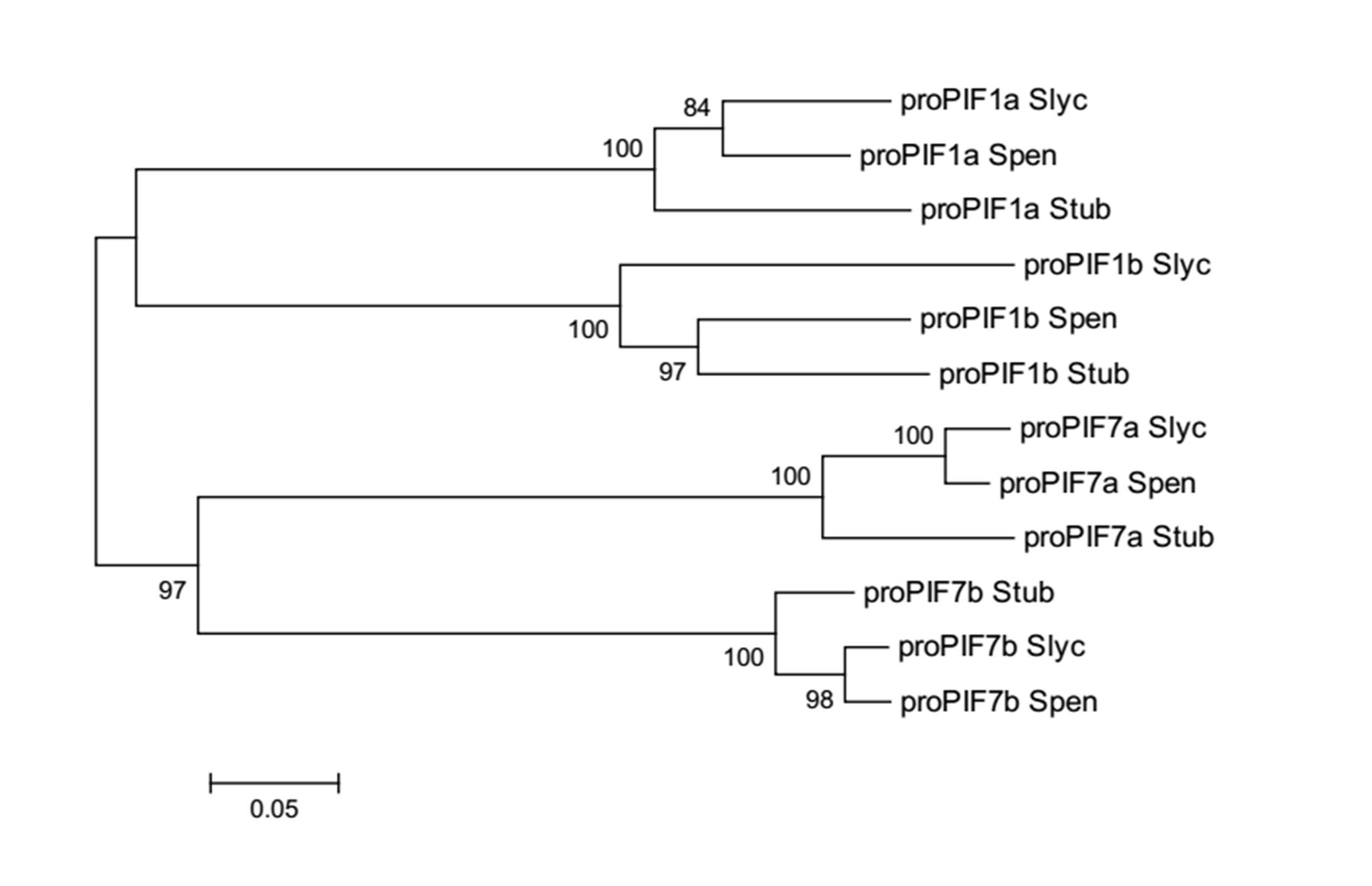

Supplement: S8 Fig — (TIF) [file pone.0165929.s008.tif]
