# Supplementary material for: Phytochrome Interacting Factors (PIFs) in Solanum lycopersicum: Diversity, Evolutionary History and Expression Profiling during Different Developmental Processes
Source: PLoS One. 2016 Nov 1;11(11):e0165929. doi: 10.1371/journal.pone.0165929 (PMC5089782; doi:10.1371/journal.pone.0165929)
Supplement: S9 Fig — The motifs identified in S7 Fig are highlighted in yellow (CArG [42]), blue (PBE-box [40]), green (CA-hybrid [41]), orange (CG-hybrid [41]) and pink (ACE-motif [41]). (PDF) [file pone.0165929.s009.pdf]

a

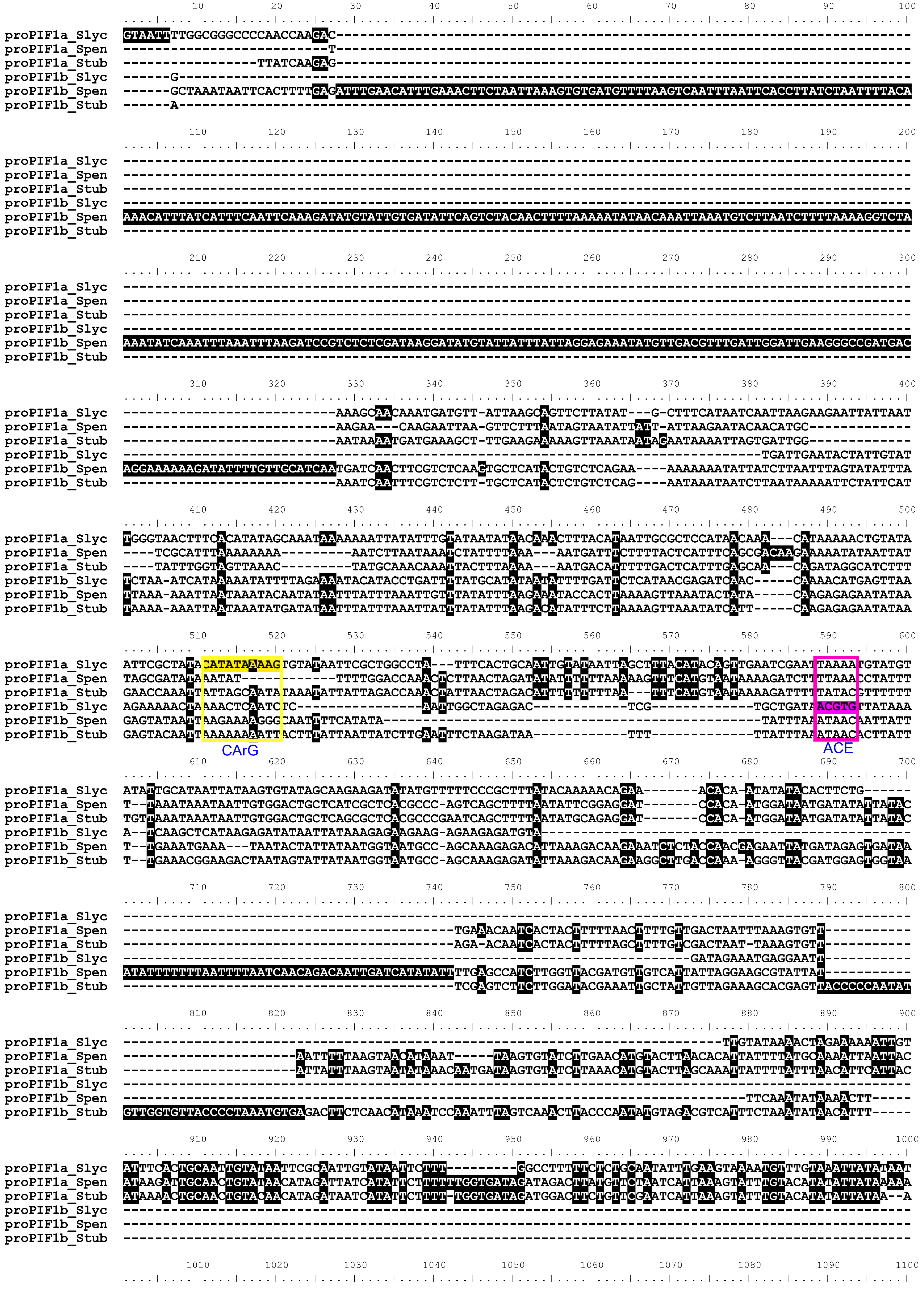

proPIF1a_Slyc
proPIF1a_Spen
proPIF1a_Stub
proPIF1b_Slyc
proPIF1b_Spen
proPIF1b_Stub
CArG
ACE

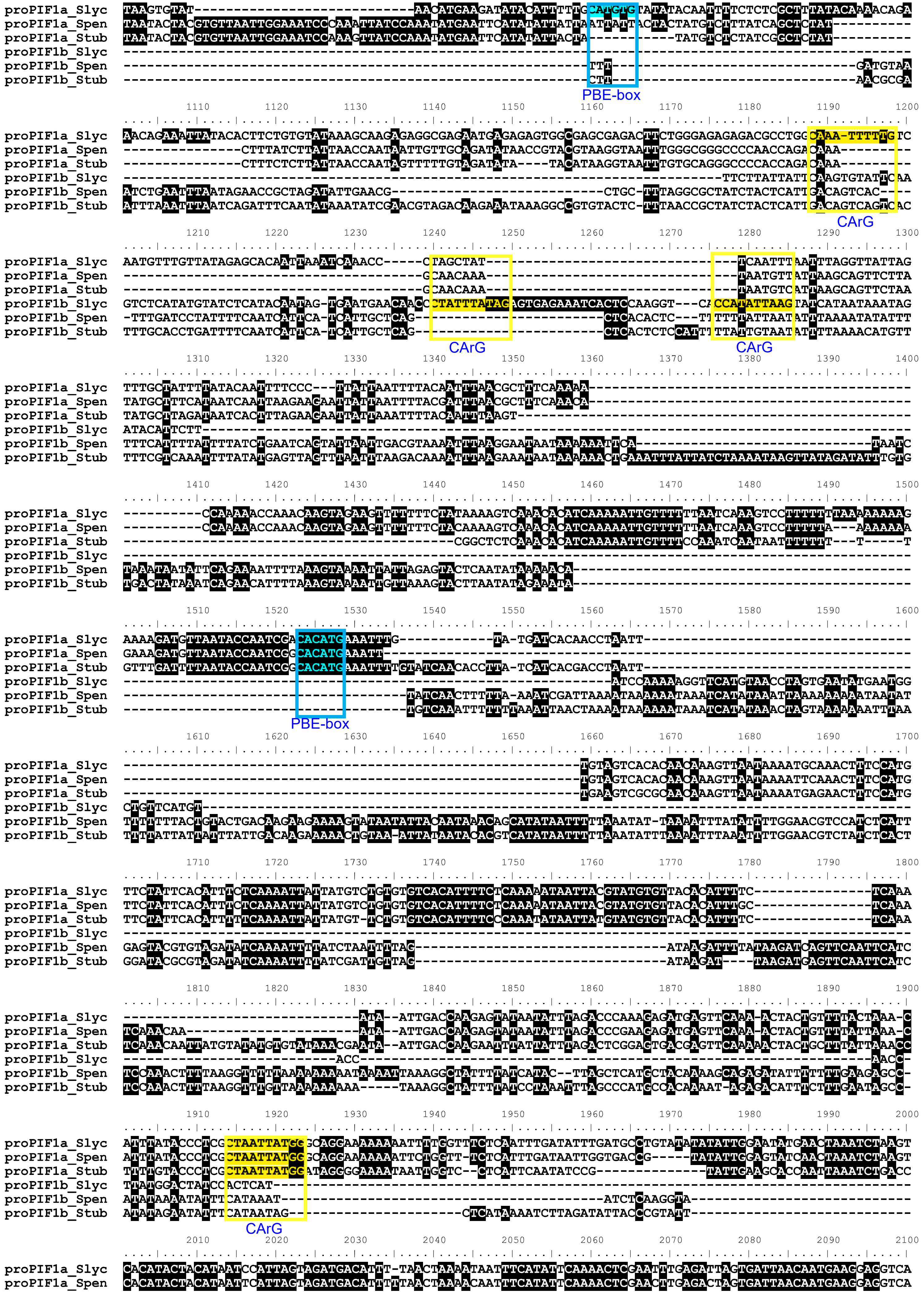
proPIF1a_Slyc
proPIF1a_Spen
proPIF1a_Stub
proPIF1b_Slyc
proPIF1b_Spen
proPIF1b_Stub
PBE-box
CArG
CArG
CArG
PBE-box
CArG

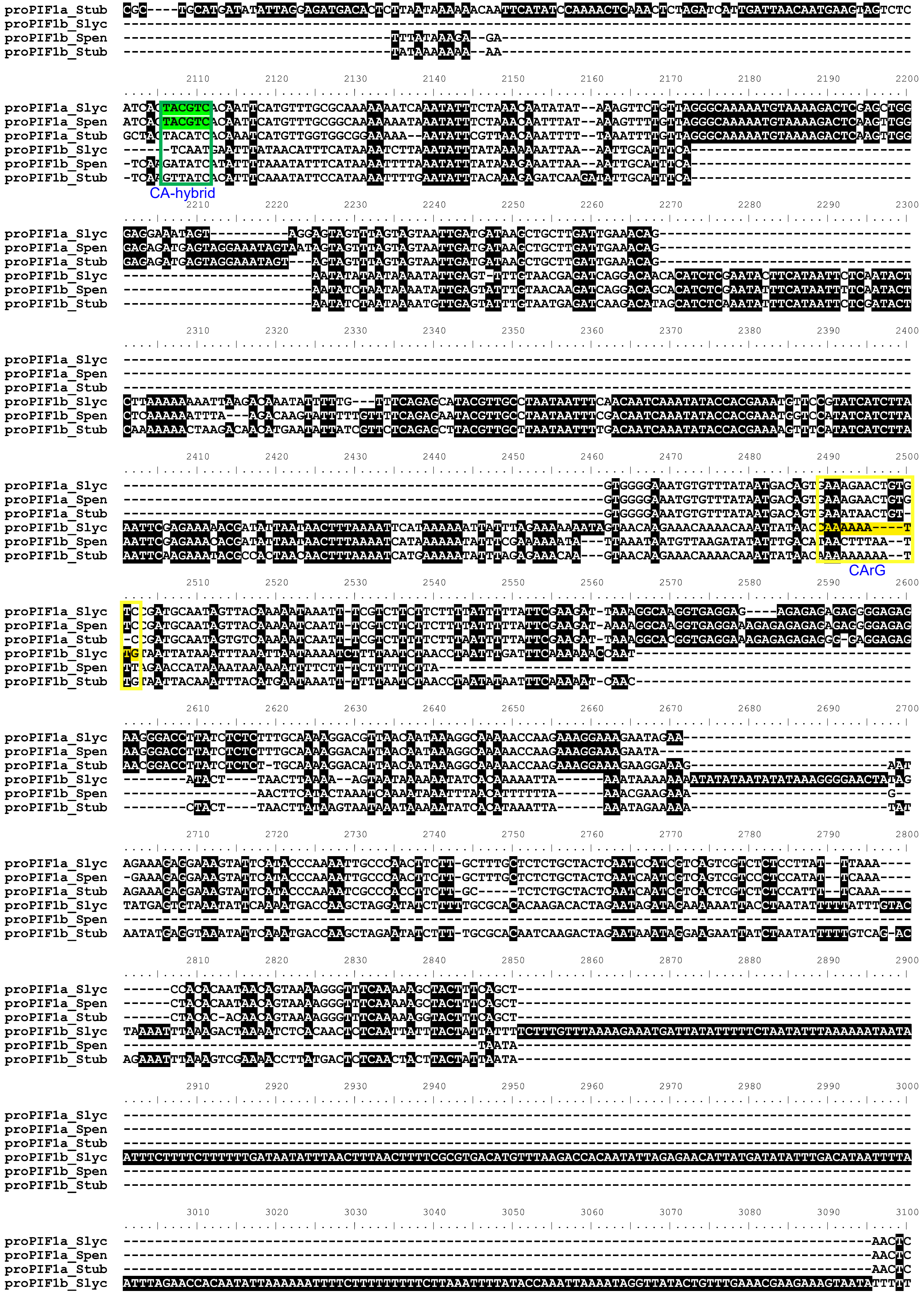
proPIF1a_Slyc
proPIF1a_Spen
proPIF1a_Stub
proPIF1b_Slyc
proPIF1b_Spen
proPIF1b_Stub
CA-hybrid
CArG

```
proPIF1b_Spen  -----------------------------------------------------------------------------------------------TTTTT
proPIF1b_Stub  -----------------------------------------------------------------------------------------------TTATC

                         3110      3120      3130      3140      3150      3160      3170      3180      3190      3200
               ....|....|....|....|....|....|....|....|....|....|....|....|....|....|....|....|....|....|....|....|
proPIF1a_Slyc  G-GAG-----------------------------------------------------------------------------------------------
proPIF1a_Spen  G-GAG-----------------------------------------------------------------------------------------------
proPIF1a_Stub  TGGAG-----------------------------------------------------------------------------------------------
proPIF1b_Slyc  A-TAAGGCCAAATACATAAACAGATCCCTAAATTTGTTGGCTTTTTTCCCTCAGGTACCTCAACTATGTCATTTTTCTATTGAATCATTGAATTATCCGT
proPIF1b_Spen  A-TAA-----------------------------------------------------------------------------------------------
proPIF1b_Stub  A-TAA-----------------------------------------------------------------------------------------------

                         3210      3220      3230      3240      3250      3260      3270      3280      3290      3300
               ....|....|....|....|....|....|....|....|....|....|....|....|....|....|....|....|....|....|....|....|
proPIF1a_Slyc  ----------------------------------------------------------------------------------------------------
proPIF1a_Spen  ----------------------------------------------------------------------------------------------------
proPIF1a_Stub  ----------------------------------------------------------------------------------------------------
proPIF1b_Slyc  AATTTATTACTTTAAAACACTGTTGGTTGATTTTGATCGGCTTTTCTATTGTAAATGCCTTCAATTGTGTTCGAATTGTAATAATTTTGATTGAAGGAAT
proPIF1b_Spen  ----------------------------------------------------------------------------------------------------
proPIF1b_Stub  ----------------------------------------------------------------------------------------------------

                         3310      3320      3330      3340      3350      3360      3370      3380      3390      3400
               ....|....|....|....|....|....|....|....|....|....|....|....|....|....|....|....|....|....|....|....|
proPIF1a_Slyc  ----------------------------------------------------------------------------------------------------
proPIF1a_Spen  ----------------------------------------------------------------------------------------------------
proPIF1a_Stub  ----------------------------------------------------------------------------------------------------
proPIF1b_Slyc  GAAGAAAAAACCGTGTTAGTCTCATTTGTTTTCCAATGTGTTCAAATGACTTAAGTAAAACACAATTATTCTCGAACATTTTCACTATCAATTGAACTAAT
proPIF1b_Spen  ----------------------------------------------------------------------------------------------------
proPIF1b_Stub  ----------------------------------------------------------------------------------------------------

                         3410      3420      3430      3440      3450      3460      3470      3480      3490      3500
               ....|....|....|....|....|....|....|....|....|....|....|....|....|....|....|....|....|....|....|....|
proPIF1a_Slyc  ----------------------------------------------------------------------------------------------------
proPIF1a_Spen  ----------------------------------------------------------------------------------------------------
proPIF1a_Stub  ----------------------------------------------------------------------------------------------------
proPIF1b_Slyc  GAATTTTGAAACAACGTATGTATCCTTTATCGATACCCCTCAAAAATTCGATTCCACATCAAACAACGGTGTTTGTTTAAACACCAAATTATGAGGGTTC
proPIF1b_Spen  ----------------------------------------------------------------------------------------------------
proPIF1b_Stub  ----------------------------------------------------------------------------------------------------

                         3510      3520      3530      3540      3550      3560      3570      3580      3590      3600
               ....|....|....|....|....|....|....|....|....|....|....|....|....|....|....|....|....|....|....|....|
proPIF1a_Slyc  ---------------------------------------------------------------------------------------------GTTGTTA
proPIF1a_Spen  ---------------------------------------------------------------------------------------------GTTGTTA
proPIF1a_Stub  ---------------------------------------------------------------------------------------------GTTGTTA
proPIF1b_Slyc  AACGATTCAATAGAAAATGACGTAGTTGAGGTACCTGAGGAAAAAAACCCAACAAATTTAGAAATCTGCTTATGTATTTGGCGTTTTTATATTAATATA
proPIF1b_Spen  ---------------------------------------------------------------------------------------------TAATATA
proPIF1b_Stub  ---------------------------------------------------------------------------------------------TAATATA
                                 CA-hybrid
                         3610      3620      3630      3640      3650      3660      3670      3680      3690      3700
               ....|....|....|....|....|....|....|....|....|....|....|....|....|....|....|....|....|....|....|....|
proPIF1a_Slyc  AAGACGCTTTTAACAGTAGCAG----CCTTTTCTGTGTGTGA----------------------------------------------------------
proPIF1a_Spen  AAGACGCTTTTAAGAATAGCAG----CCTTTTCTG----TGA----------------------------------------------------------
proPIF1a_Stub  AAGACGTTTTTAACATTAAGCAGCCGCCTTTTGTG----TGA----------------------------------------------------------
proPIF1b_Slyc  AATAAATTAAAAATATTTGAGG----CTAGCTAAGTCGGTAGCAAGTGAAAACTAAAACACAGAGATATTTATAACAAGTCAAAAACTACTAAAACAGTT
proPIF1b_Spen  AATAAATTAATAATATTTGAGG----CTAGCTAAGTCGGTAGCAAGTGAAAACTAAAACACAGATATAT-TATAACAAGTCAAAAACTACTAAAACAGTT
proPIF1b_Stub  AATAAAATAATAATATTTCGAG----CTAGCTAAGTCGGTAGCAAGTCAAAATTAAAACACACAGATAT-TATAGCAAGTCAAAAACTACTAAAACAATT

                         3710      3720      3730      3740      3750      3760      3770      3780      3790      3800
               ....|....|....|....|....|....|....|....|....|....|....|....|....|....|....|....|....|....|....|....|
proPIF1a_Slyc  ----------GCTTTTGATT---------TATGGGCGGAGCTAAT-AA--GCTCCAGTGTTATGGTGAGTTTGGGACTGCTTCTTGCGGCTGTGCCTGAC
proPIF1a_Spen  ----------GCTTTTGATT---------TATGGGCGGTGCTAAATAA--GCTCCAGTGTTATGGTGAGTTTGGGACTGCTTCTTGCGGCTGTGCCTGAC
proPIF1a_Stub  ----------GCTTTTGATTTCGTAGATTTATGGGCGGTGTTAATAAGCTGCTCCAGTGTTATGGTGAGTTTGGGACTGCTTGTTGCGGCGGTGCCTGAC
proPIF1b_Slyc  CTCATGGAGAGCAAAACTCAAAATGAGTT-----------------------------------------------------------------------
proPIF1b_Spen  CGA-------CCAAAACTCAAAACGAGTT-----------------------------------------------------------------------
proPIF1b_Stub  CTCACA---ACAGCAGCTCAAAACGAGTT-----------------------------------------------------------------------

                         3810      3820      3830      3840      3850      3860      3870
               ....|....|....|....|....|....|....|....|....|....|....|....|....|....|.
proPIF1a_Slyc  CAATTTTGAATTATTTTTGCTT-TTTTTTTTGG----------GAGATTTGAGCTGCCTACGTCACG----
proPIF1a_Spen  CAATTTTGAATTATTTTTGCTTTTTTTTTTTGG----------GAGATTTGAGCTGCCTACGTTACG----
proPIF1a_Stub  CAATTTTGA-----------TTTTTTTATTTGG----------GAGATTTGAGCTGCCTACGTCACG----
proPIF1b_Slyc  -----CATG-----------AATAAATCTCAAGTGAGTTTCTGATGATATAATTCAATTAAACTCACAAAG
proPIF1b_Spen  -----CATG-----------AATAAATCTCAAGTGAGTTTCTGATGATATAATTGAATTAAACTCACAAAG
proPIF1b_Stub  -----CATA-----------TAAAAGAAGAACA----------AATATTTAATTGAATTAAATCACAAG--
                                                                         CA-hybrid
```

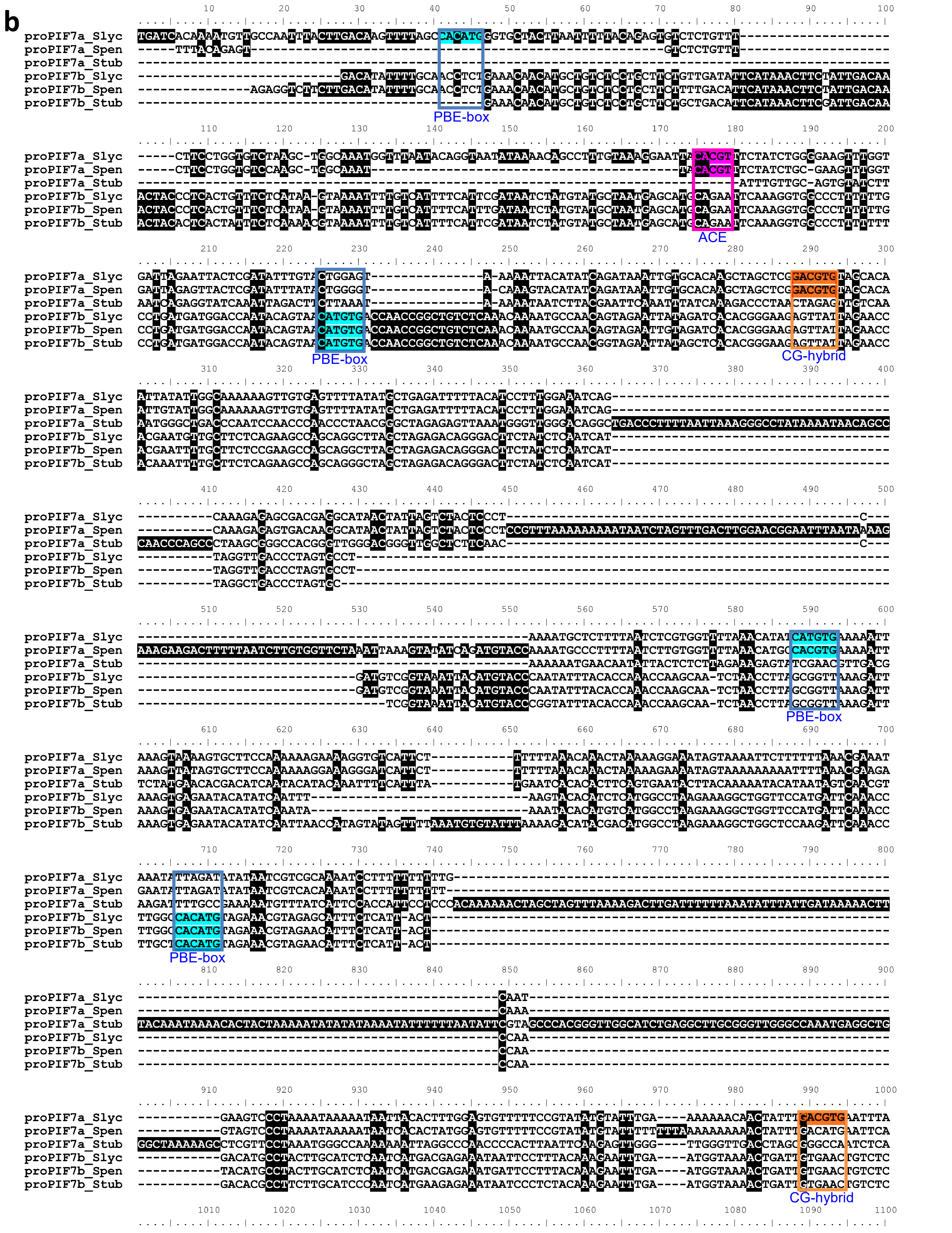

b
proPIF7a_Slyc
proPIF7a_Spen
proPIF7a_Stub
proPIF7b_Slyc
proPIF7b_Spen
proPIF7b_Stub
PBE-box
ACE
CG-hybrid

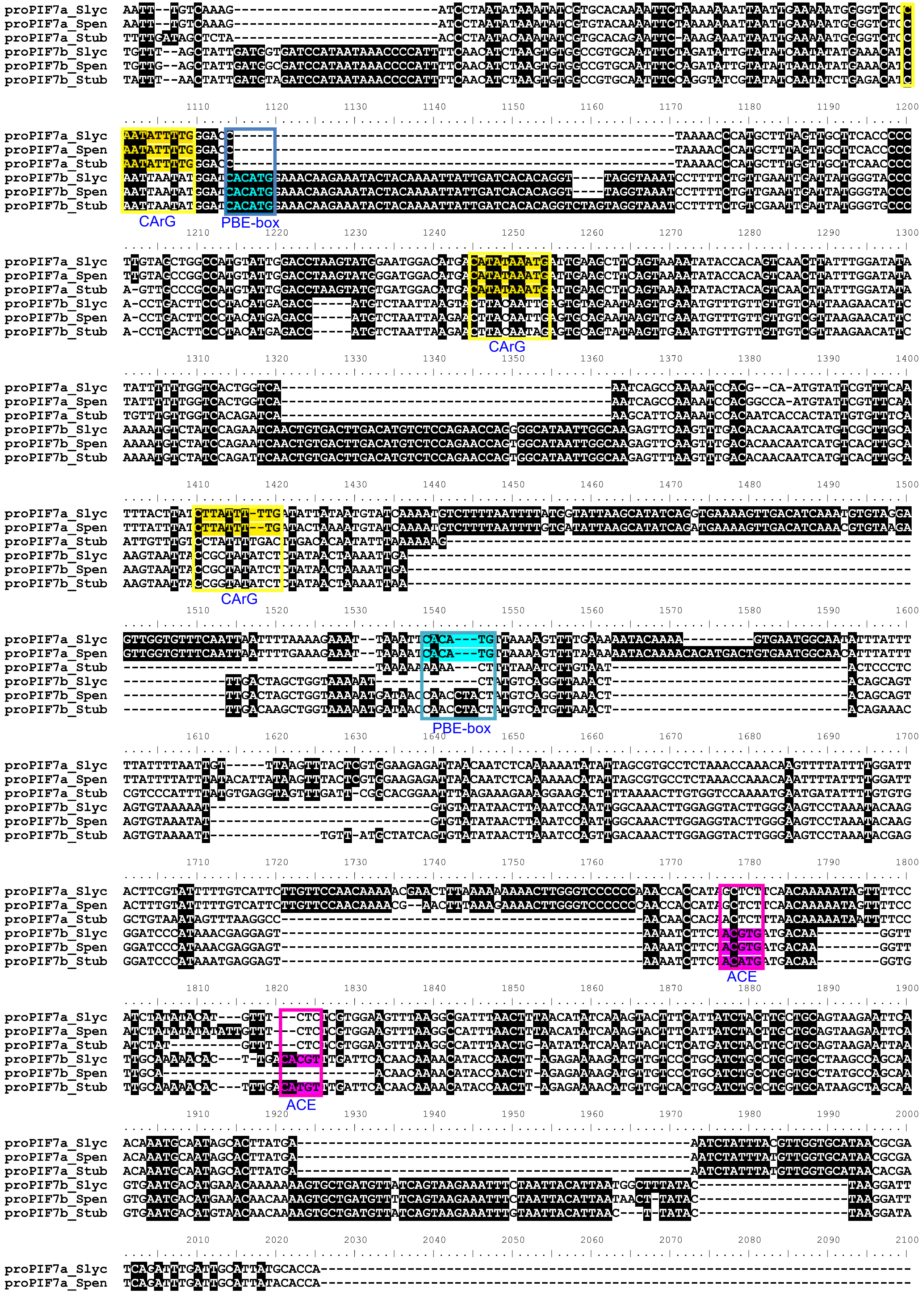

CArG
PBE-box
CArG
CArG
PBE-box
ACE
ACE

```
proPIF7a_Stub  TCAAATTTGATTGCATTATGCACCAGTTAATATGATAGGAACTAAATTCAGGCTTAAGAAAATAGATTTTGAATCATCTTTCCTTAAGGAATATCGTGAT
proPIF7b_Slyc  CCAAAATCTAATCTAACAGCACTTG---------------------------------------------------------------------------
proPIF7b_Spen  CCAAAATTTAATCTAACAGCACTTG---------------------------------------------------------------------------
proPIF7b_Stub  CCAAATTTTAATCTAACAGC----G---------------------------------------------------------------------------

                        2110      2120      2130      2140      2150      2160      2170      2180      2190      2200
               ....|....|....|....|....|....|....|....|....|....|....|....|....|....|....|....|....|....|....|....|
proPIF7a_Slyc  -------------------------ACTTGATTTTTAACCCAAAAGTCATACGTATAAAAAA-ACAAAGTTGAGCGACTTTCTGTCAGA-AAAATTCATA
proPIF7a_Spen  -------------------------ACTTGATTTTTAACCCAAAAGTCATACTTATAAAAAAAATAAAGTTGAGCGACTTTCTGTTAGA-AAAATCA---
proPIF7a_Stub  AACAAACTCTCCAGAAAGTCACTCAACTTAATTTTTAACCCAAAAGTCATACGTATAAAAATC--ATAGTTGAGCGACGTTTAGTCTAA-AAAGTTCATA
proPIF7b_Slyc  -------------------------ATTAGAACTTTATACCAATGTTTATACGA-----AATC--ATGGATGAGAATGACAAAAGATAAACCACAAATTA
proPIF7b_Spen  -------------------------ATTAGAACTTTATACCAATGTTTATACGA-----AATC--ATGGATGAGAATGACAAAAGATA-ACCACAAATTA
proPIF7b_Stub  -------------------------CTTAGAACTTTATTCTAATGTTTATACGA-----ATTC--AGGGATGAAAGTGACAAAAGATA-ACTACAAAGTA

                        2210      2220      2230      2240      2250      2260      2270      2280      2290      2300
               ....|....|....|....|....|....|....|....|....|....|....|....|....|....|....|....|....|....|....|....|
proPIF7a_Slyc  ATTGAGTGACAATATGCGATGTTATCTCTGATTGAGGGCATCTCTCAAATTTTAAGGCCCCATCCTAGCAGGTTGTTGTTCAACATT--AAATTCGATGG
proPIF7a_Spen  ---------------GTGATATTATTTCTGATTAAGGGCATCTATCAAATTTTAAGGCCCCATTCTAGCAGGTTGTTGTTCAACATT--AAATTCGATGG
proPIF7a_Stub  GTTGAGTGACCATCTGTGAT-TAATCTCTGATTGAGGGCATCTCTCAAATTTTAAGTCCCCATCCTAGCAGGTTGTTGTTCAGACATTAAATTTCGATGG
proPIF7b_Slyc  CCAGCCCCACCTCATCTTAT-GAAGTCGGCATACCTTCTATGTGATTGAAATTAA------------------------------------TTATATTTT
proPIF7b_Spen  CCAGCCCCACCTCATCTTAT-GAAGTCGGCATAACTTCTATGTGATTGAAATTAA------------------------------------TTATATTTT
proPIF7b_Stub  CCAGCCCCACCTCATCTTAT-GAAGTCGGCATAACTTCTATGTGATTGAAATTAA------------------------------------TTATATTTT

                        2310      2320      2330      2340      2350      2360      2370      2380      2390      2400
               ....|....|....|....|....|....|....|....|....|....|....|....|....|....|....|....|....|....|....|....|
proPIF7a_Slyc  C-TCCCGCCCTCTAAAAGCTTGTGGTGGAGGAATAGTACTAGTACTATTTATGTAGGTCCAAAAACAGTAAAACACTAG----AAATGTAAATGCATTGA
proPIF7a_Spen  C-TCCCGCCCTCTAAAAGCTTGTGGTGGAGGAATAGTATTAGCACTATATATGTAGGTCCAAAAACACTGAAACACTAG----AAATGTAAATGCATTGA
proPIF7a_Stub  A-TCCCGCCTTCTAAAAGCTTGTGGCGGAGGAATAGTAGTAGTACTATTTATGTAGGTCCAAAAACAGTGAAACACTAG----AAATCTAAATGCATTGA
proPIF7b_Slyc  CTCAAAATGCTGTCAATTTATTT-------GAATAGATGTTGCATCACTAGTCAGAGCAACCAAAGTCAGATAGAGAGGCGGCAAATTTGAACGCATTGA
proPIF7b_Spen  CTTAAAGTACTATCAATTTATTT-------GAATGGATGTTGCATCAATAGTCAGAGCAACCAAAGTCAGATAGAGAGGCGGCAAATTTGAACGCATTGA
proPIF7b_Stub  T-TAAAATGTTGTCAATTTATTT-------GAATAGTTGTTGCATCAATAGTCAGAGCAACCAAAGTCAGATAGAGAGGTGGCAAATTTGAACGTATTGA

                        2410      2420      2430      2440      2450      2460      2470      2480      2490      2500
               ....|....|....|....|....|....|....|....|....|....|....|....|....|....|....|....|....|....|....|....|
proPIF7a_Slyc  GTGCTTGTAACCCCACCTAACATCAACAAAATCTTAATTGGAATGAAGGTA-------------------------------------------------
proPIF7a_Spen  GTGCTTGTAACCCCACCTAACATCAACAAA-TCTTAATTGGAATGAAGGTA-------------------------------------------------
proPIF7a_Stub  GTGCTTGTAACCCCACCTAACTTCAACAAA-TCTTAATTGGAATGAAGGTA-------------------------------------------------
proPIF7b_Slyc  ATGTTTTTAAACCCATCAAAATGGAAA-TA-TCTTTGTTTGAAGTTACTAATTACGATGAAGAAATCAGTTCCTGATCTTTAGGTGGATAAAAATTTTGC
proPIF7b_Spen  ATGTTTTTAAACCTATCAAAAAGGAAAATA-TCTTTGTTTGAAGTTACTAATTACAATGAAGAAATCAGTTCCTGATCTTTAGGTGGATATAGATTTTGC
proPIF7b_Stub  ATGTTTTTAACCCCATCAAAAAGGAAAATA-TCTTTGTTGGAAGTTACTAATTACAATGAAGAAATCAGTTCCTGATCTTTAGGTGGATAAAATTTTTGC

                        2510      2520      2530      2540      2550      2560      2570      2580      2590      2600
               ....|....|....|....|....|....|....|....|....|....|....|....|....|....|....|....|....|....|....|....|
proPIF7a_Slyc  ------------------------------------------AGAAGAAAT--CACATATATGAAGAGATATCCATTTGAAATCCAGGCTTTCTTGCTGT
proPIF7a_Spen  ------------------------------------------AGAAGAAAT--CACATATATGAAGAGATATCCATTTGAAATCCAGGCTTTCTTGCTGT
proPIF7a_Stub  ------------------------------------------AGAAGAAACTGCACATATATGAAGAGATATCCATTTGAAATCCAGGCTTTCTTGCTAT
proPIF7b_Slyc  TCTTTCGTTCTTTAGAGTTTCTATCATCAAAGAAAAAGAGTAAAACAGATACACTAAGATGCCAAAACTTATCCATTTGAAATCCAGGCTTTTAT----T
proPIF7b_Spen  TCTTTCATTCTTTAGAGTT-CTATCATCAAAGAAAAAGAGTAAAACAGATACACTAAGATGCCAAAACTTATCCATTTGAAATCCAGGATTTTAT----T
proPIF7b_Stub  TCTTTCATTCTTTAGAGTT-CT--------------------------ATCATCTAAGATGCCAAAACTTATCCATTTGAAATCCAGGCTTTTAT----T

                        2610      2620      2630      2640      2650      2660      2670      2680      2690      2700
               ....|....|....|....|....|....|....|....|....|....|....|....|....|....|....|....|....|....|....|....|
proPIF7a_Slyc  GTCACATGTTGTGTGTTTCAGTATATAATTGGTCATTTGAATGCGACAATTCAAAAAGGGGACCAGAAAGCAATAAGAATAATTAAGTTTTTAG------
proPIF7a_Spen  GTCACATGTTGTGTGTTTCAGTATATAATTGGTCATTTGAATGCGACGATTCAAAAAGGGGACCAGAAAGCAATAAGAATAATTAAT-TTTTAG------
proPIF7a_Stub  GTCACATGTTGTGTGTTTCAGTATATAATTAGTCATCTGAATGCGACAATGCAAAAAGGGGACCAGAAAGCAATAAGAATAATTAA-TTTTTAG------
proPIF7b_Slyc  GCTACAACACACAGTGTGCATACATTAAATATTCAGCTAAATATGACAATCCTGTAGGGGACCAGAAGATACTGAAAGACATTTTTAGTATAAAAGAGGG
proPIF7b_Spen  GCTACAACACATAGTGTGCATACATTAAATATTCAGCTAAATATGACAATCCTGTAGGGGACCAGAAGATACTGAAAGACATTTTTAGTATAAAAGAGGG
proPIF7b_Stub  GCTACAACACATAGTGTGCATACATTAAATATTCAGCTAAATATGACAATCCTGTAGGGGACCAGAAGATACTGAAATACGTTTTTAGTATAAAAGAGGG
                 PBE-box
                        2710      2720      2730      2740      2750      2760      2770      2780      2790
               ....|....|....|....|....|....|....|....|....|....|....|....|....|....|....|....|....|....|....|..
proPIF7a_Slyc  -------------TATATAAAACTGAGAAGAAGCAGAGAGTTCAACATTCGGGAACAGAGAAAAAGAATAGATTACTCAATAATAATATTAATCATC
proPIF7a_Spen  -------------TATATAAAACTGAGAAGAAGCAGAGAGTTCAACATTCGGGAACAGAGAAAAAGAATAGATTACTCAATAATAATATTAATCATC
proPIF7a_Stub  -------------TATATAAAACAGAGAATAAGCAGA--GTTCAACATTTGGGAACAGAGAAAAGGAATAGATTACTCAATAATCATATTAATCATC
proPIF7b_Slyc  AAAGCCAACTCAATATATTAGGACTGGAAGTAACTGAGAAATTAAGAGAGTAGACCCTATCAAAGGCATCAAC------------------------
proPIF7b_Spen  AAAGCCAACTCAATATATTAGGACTGGAAGTAACTGAGAAATTAAGAGAGTAGACCCTATCAAAGGCATCAAC------------------------
proPIF7b_Stub  AAAGCCAACTCAATATATTAGGACTGGCAGTAACTGAGAACTTATAAGGGTAGACCCTATCAAAGGCATCAAC------------------------
```
